# The inflammasome-activating poxvirus peptide IAMP29 promotes antimicrobial and anticancer responses

**DOI:** 10.1038/s12276-024-01339-3

**Published:** 2024-11-01

**Authors:** Taylor Roh, Wonhyoung Seo, Minho Won, Woo Seok Yang, Asmita Sapkota, Eun-Jin Park, Sung-Ho Yun, Sang Min Jeon, Kyung Tae Kim, Bomi Lee, Gyoungah Ryu, Sang-Hee Lee, Jung-Min Shin, Hyo Jung Shin, Young Jae Kim, Young Lee, Chaeuk Chung, Ik-Chan Song, Hyun Kyu Song, Eun-Kyeong Jo

**Affiliations:** 1 Department of Microbiology, Chungnam National University College of Medicine, Daejeon, Republic of Korea; 2 Infection Control Convergence Research Center, Chungnam National University College of Medicine, Daejeon, Republic of Korea; 3 Department of Medical Science, Chungnam National University College of Medicine, Daejeon, Republic of Korea; 4 Brain Korea 21 FOUR Project for Medical Science, Chungnam National University College of Medicine, Daejeon, Republic of Korea; 5 Department of Biochemistry, Chungnam National University College of Natural Sciences, Daejeon, Republic of Korea; 6 Department of Life Sciences, Korea University, Seoul, Republic of Korea; 7 Bio-Chemical Analysis Team, Korea Basic Science Institute, Cheongju, Republic of Korea; 8 Center for Research Equipment, Korea Basic Science Institute, Cheongju, Republic of Korea; 9 Department of Dermatology, Chungnam National University College of Medicine, Daejeon, Republic of Korea; 10 Department of Anatomy and Cell Biology, Chungnam National University College of Medicine, Daejeon, Republic of Korea; 11 Brain Research Institute, Chungnam National University College of Medicine, Daejeon, Republic of Korea; 12 Division of Pulmonary and Critical Care Medicine, Department of Internal Medicine, Chungnam National University Hospital, Daejeon, Republic of Korea; 13 Division of Hematology/Oncology, Department of Internal Medicine, Chungnam National University College of Medicine, Daejeon, Republic of Korea

**Keywords:** Antimicrobial responses, Cancer therapy, Inflammasome

## Abstract

Poxviruses are implicated in a variety of infectious diseases; however, little is known about the molecular mechanisms that underlie the immune response during poxvirus infection. We investigated the function and mechanisms of the monkeypox virus envelope protein (A30L) and its core peptide (IAMP29) during the activation of innate immune responses. The A30L protein and its core peptide, IAMP29 (a 29-amino-acid inflammasome-activating peptide encompassing His40 to Asp69 of A30L), strongly activated the nucleotide-binding oligomerization domain, leucine rich repeat and pyrin domain-containing 3 (NLRP3) inflammasome by inducing the production of mitochondrial reactive oxygen species in human monocytes. Specifically, IAMP29 triggered metabolic reprogramming toward glycolysis and interacted with pyruvate kinase M isoforms (PKM1 and PKM2), thus activating the NLRP3 inflammasome and interleukin (IL)-1β production in human monocytes and murine macrophages. In human primary monocyte-derived macrophages, IAMP29-induced inflammasome activation promoted an antimicrobial response to rapidly growing non-tuberculous mycobacteria. Furthermore, IAMP29 exhibited cytotoxic activity against leukemia cells, which was mediated by pyroptosis and apoptosis. These findings provide insights into the immunological function of the poxvirus envelope peptide and suggest its therapeutic potential.

## IAMP29 peptide triggers immune response and cancer cell death

Poxviruses, a group of viruses causing diseases like smallpox and mpox, can manipulate our immune system, but the exact process is not fully understood. This study reveals that a specific poxvirus protein, IAMP29, can stimulate our immune response. It does this by causing NLRP3 inflammasome activation and boosting our body’s defenses against infections and cancer. The research, which involved experiments with human immune cells and mice, found that IAMP29 increases the production of inflammation-causing molecules and strengthens the immune system’s ability to combat fast-growing non-tuberculous mycobacteria (bacteria that do not cause tuberculosis and leprosy) and leukemia cells. The study suggests that IAMP29 could be used as a treatment for these bacterial infections and certain cancers, providing a significant insight into how poxviruses interact with our immune system. This could pave the way for new treatments for infectious diseases and cancer.

This summary was initially drafted using artificial intelligence, then revised and fact-checked by the author.

## Introduction

Poxviruses, which are members of the Poxviridae family, are double-stranded DNA viruses with large, brick- or oval-shaped structures and genomes of 130 to −300 kilobases (kb). They exhibit a wide host range and exclusively replicate in the cytoplasm of infected cells^1,2^. Among the members of the Poxviridae family, only four genera (*Orthopoxvirus*, *Parapoxvirus*, *Molluscipoxvirus*, and *Yatapoxvirus*) of the Chordopoxvirinae subfamily are capable of infecting humans^2^. Several poxviruses, such as variola virus, monkeypox (mpox) virus (MPXV), vaccinia virus, and molluscum contagiosum, have caused substantial outbreaks, primarily due to their efficient transmission between humans^1,2^. Although the smallpox eradication program led by the World Health Organization (WHO) was successful more than 25 years ago, concern exists regarding the potential re-emergence and spread of related pathogenic poxviruses in the human population^1,2^. Indeed, mpox has caused substantial morbidity and mortality, particularly among pregnant women, immunocompromised individuals, and children^1,3,4^. Nevertheless, little is known about the viral effectors/modulators that interfere with or dysregulate host immune functions.

Poxviruses encode an array of self-defense proteins that counteract host innate and adaptive immune responses^5,6^. These proteins target immune molecules involved in processes such as innate immune responses, cytokine production, and inflammatory signaling^5,7^. The interplay between viral proteins and host responses may influence host tropism, protective immune responses, and disease progression during poxvirus infections. Inflammasomes are cytosolic protein complexes that mediate the activation of potent inflammatory mediators, such as interleukin (IL)-1β. Among the inflammasomes reported thus far, the NOD-, LRR-, and pyrin domain-containing protein 3 (NLRP3) inflammasome has been the most extensively studied. A two-step process is required for IL-1β secretion after activation of the NLRP3 inflammasome complex. Signal 1 and the priming steps are mediated by the nuclear factor (NF)-κB pathway, cytokine signaling, and posttranslational modification of inflammasome components. Signal 2 (leading to the assembly of the NLRP3 inflammasome complex) is activated by several stimuli, including mitochondrial dysfunction, ion flux (e.g., K^+^ efflux), and lysosomal damage^8,9^. The NLRP3 inflammasome orchestrates protective and pathological responses, depending on the context, in various human diseases, including viral infections and autoinflammatory and autoimmune disorders^10,11^. However, whether poxvirus protein components manipulate the host inflammasome machinery is unclear.

The activation of the NLRP3 inflammasome is essential for suppressing bacterial invasion and dissemination and for enhancing the clearance of pathogens such as *Clostridium perfringens*^12^, *Pseudomonas aeruginosa*^13^, and invasive pneumococci^14^. Non-tuberculous mycobacteria (NTMs), which are distinct from *Mycobacterium tuberculosis* (*M. tuberculosis*) and *Mycobacterium leprae*, are environmental bacteria that have become increasingly prevalent worldwide. Some of the more than 190 species of NTMs cause infections at several body sites, affecting both immunocompromised and immunocompetent individuals^15,16^. The *Mycobacterium abscessus* (*M. abscessus*) complex is a rapidly growing group of NTMs that can be identified within 7 days, whereas the *Mycobacterium avium* (*M. avium*) complex requires up to 12 weeks for detection^16^. The *M. abscessus* complex comprises three subspecies: *M. abscessus* subsp. *abscessus* (Mabc), *M. abscessus* subsp. *bolletii* (Mboll), and *M. abscessus* subsp. *massiliense*^17^. Two morphotypes of *M. abscessus* are observed, smooth or rough colonies, depending on the presence of surface glycopeptidolipids^18^. These NTM species exhibit high levels of antimicrobial resistance and pose challenges for treatment. Mabc is one of the most antibiotic-resistant mycobacterial species, for which few therapeutic options are available^18^. Considerable effort has been dedicated to developing cutting-edge strategies that combat antibiotic resistance in NTMs, with a focus on novel antimycobacterial compounds^19^. Activation of the NLRP3 inflammasome restricts the intracellular growth of Mabc in human monocytes/macrophages^20^. In this context, the identification of novel agents that promote NLRP3 inflammasome activation and enhance innate host defense against Mabc is an important research objective.

Pyroptosis, which involves inflammasome activation and the formation of cell-membrane pores in a gasdermin (GSDM) family-mediated manner, has implications for the immune system and cancer immunotherapy^21–23^. Considering that inflammasome activation is important for eliminating cancer cells in response to cellular damage, pyroptosis modulation has therapeutic potential for cancer^21,24^. Substantial effort has focused on identifying targets for anticancer therapy, with the goal of improving the selective eradication of tumor cells while preserving the integrity of normal cells. Naturally occurring compounds, such as polyphenols, are cytotoxic to tumor cells but not normal cells^25^. However, their inflammasome-activating abilities are unclear.

Here, we report that MPXV envelope protein (A30L) triggers the expression of a set of host inflammatory genes, as revealed by a transcriptomic analysis of human peripheral blood mononuclear cells (PBMCs). A30L and its core peptide (IAMP29), a 29-amino acid inflammasome-activating peptide (His40 to Asp69 of A30L), induced IL-1β production and activated the NLRP3 inflammasome in several types of immune cells, including PBMCs, differentiated THP-1 cells, human primary monocytes, and murine macrophages. Additionally, IAMP29 induced metabolic reprogramming toward glycolysis and interacted with two pyruvate kinase M isoforms (PKM1 and PKM2), leading to the activation of the NLRP3 inflammasome in human immune cells. Importantly, IAMP29-driven inflammasome activation enhanced antimicrobial responses against rapidly growing NTMs in human primary monocyte-derived macrophages (MDMs), murine bone marrow-derived macrophages (BMDMs), and peritoneal macrophages (PMs). Furthermore, IAMP29 triggered the pyroptotic and apoptotic death of leukemia cells both in vitro and in vivo, demonstrating its potential as an antileukemic agent. Therefore, the novel poxvirus peptide IAMP29 has therapeutic potential for NTM infections and cancers.

## Materials and methods

### Ethical approval and consent to participate

This study was performed in compliance with the Declaration of Helsinki and was approved by the Institutional Research and Ethics Committee of Chungnam National University Hospital (Daejeon, South Korea; CNUH IRB-2020-07-082 and CNUH IRB-2018-08-013-012). The participants provided informed consent at the time of enrollment. The treatment of the mice was conducted in compliance with the guidelines established by the Institutional Animal Care and Use Committee of the Chungnam National University School of Medicine (CNUH-IA0010-00 and 202109A-CNU-180), as well as the regulations established by the Korean Food and Drug Administration.

### Materials

The reagents, recombinant proteins, and antibodies used in this study are described in detail in the Supplementary Information.

### Cell preparation and culture

BMDMs and PMs were obtained from 8-week-old C57BL/6 wild-type female mice (Samtako Bio, Osan, Korea) and cultured. Blood samples were obtained from healthy donors in 10-mL heparinized venous blood tubes. Human PBMCs were isolated using Lymphoprep™ (STEMCELL Technologies Inc., Vancouver, BC, Canada) in accordance with the manufacturer’s instructions. CD14^+^ monocytes were prepared using a CD14 MicroBeads MACS kit (Miltenyi Biotec, Bergisch Gladbach, NRW, Germany). Detailed information on the cell preparation is provided in the Supplementary Information.

### Lentiviral shRNA transduction

Lentiviruses were produced by transfecting HEK293T cells (ATCC, Manassas, VA, USA; CRL-11268), which were seeded in 6-well plates the previous day. For lentiviral shRNA transduction, human primary monocytes cultured in 48-well plates were transduced with a lentiviral vector. Detailed procedures for the preparation of lentiviral shRNAs and knockdown vectors are provided in the Supplementary Information.

### RNA-seq analysis

RNA integrity and quality were assessed using an Agilent 2100 Bioanalyzer (Agilent Technologies, Amstelveen, the Netherlands). RNA was quantified using an ND-2000 spectrophotometer (Thermo, Inc., Waltham, MA, USA). cDNA libraries were constructed using the QuantSeq 3′ mRNA-Seq Library Prep Kit (Lexogen, Wien, Austria). A NextSeq 500 instrument was used to conduct high-throughput sequencing (Illumina, San Diego, CA, USA). The human GRCh 38 reference genome was used to map the remaining reads with Bowtie2. Bedtools software was used to calculate gene read counts, and EdgeR was used for TMM normalization. Normalized data were used to generate a heatmap and a volcano plot with the pheatmap (version 1.0.12) and ggplot2 (version 3.4.4) packages in R (version 4.1.3), respectively. Pathway enrichment analysis was performed using gene set enrichment analysis (GSEA) software (version 4.3.2).

### Modeling

The predicted models of the MPXV A30L protein were obtained using AlphaFold2^26^. The results were confirmed using RoseTTAFold^27^. The top five ranked models and two domain architectures were based on the predicted alignment errors (Fig. 3b). PyMOL software (https://pymol.org/) was used to visualize the models.

### Circular dichroism spectroscopy

Circular dichroism (CD) spectra were recorded at 4 °C on a J-1100 spectropolarimeter (Jasco) equipped with a thermoelectric temperature controller. The IAMP29 peptide (100 μM) was prepared in 10 mM potassium phosphate and 50 mM ammonium sulfate (pH 7.3). Spectra representing the averages of five scans from 250 to 185 nm were measured in a 1 mm path length cuvette using a step size of 0.5 nm and a 2 s signal averaging time. The spectra were baseline-corrected by subtracting the reference spectrum of the buffer. The secondary structures were calculated using the BeStSel server (https://bestsel.elte.hu/).

### Immunoprecipitation (IP) and IP-based LC‒tandem mass spectrometry (IP‒MS)

The cells were lysed in cold M2 lysis buffer for IP and IP–MS. Immunocomplexes were isolated from fractionated lysates using Anti-Flag® M2 affinity gel. The immunocomplexes were separated on 4‒12% SDS‒PAGE gels and subjected to western blotting with PKM1 (Cell Signaling Technology, Danvers, MA, USA, #7067) or PKM2 (Cell Signaling Technology, #4053) antibodies or visualized by staining with InstantBlue® Coomassie Protein Stain (Abcam, Cambridge, UK, ab119211) for liquid chromatography–tandem mass spectrometry (LC‒MS/MS). In-gel digestion and the LC‒MS/MS analysis were subsequently performed. The process is described in detail in the Supplementary Information.

### Bacterial infection and colony-forming unit (CFU) assay

Smooth strains of Mabc- or Mboll-infected cells (human MDMs and murine BMDMs) were lysed and analyzed to determine the number of bacteria that survived within cells. The detailed process is provided in the Supplementary Information.

### Cell proliferation/cytotoxicity assay

THP-1, HL-60, KG-1α, and K562 Red F-luc bioluminescent tumor (K562-luc) cells were seeded in 96-well plates at 70% confluence; viability assays were performed using a CCK-8 assay kit (Dojindo, Kumamoto, Japan, #CK04) or LDH Assay Kit-WST (Dojindo, 347-91751). Absolute OD values were translated to percent viability relative to the vehicle control.

### Animal experiments with tumor xenografts

For the analysis of animal models with the bioluminescent in vivo imaging system (IVIS), we used K562-luc cells. K562-luc cells were intravenously injected into the tail vein of NOD/SCID/Il2rg null (NSG) mice (GHbio, Daejeon, South Korea, Nod/scid-IL2Rg^em1^). IAMP29 was intraperitoneally administered once every 2 days beginning 7 days after the confirmed engraftment of K562-luc cells. The tumor burden was assessed using the IVIS system once weekly until 4 weeks after the transplantation of K562-luc cells.

### Poly(d,l-lactic-co-glycolic acid) nanoparticle (PLGA NP) production and characterization

PLGA NPs were acquired from Nanoglia (Daejeon, South Korea). All reagents and solvents were of analytical grade. PLGA (Purasorb® PDLG 5002A, Corbion N.V.) NPs were prepared as previously described^28^. The process is described in detail in the Supplementary Information.

### Transmission electron microscopy

The samples were dehydrated in an ethanol and propylene oxide series, successively fixed with 3% glutaraldehyde and 1% osmium tetroxide on ice for 1 h, and then cleaned with 0.1 M cacodylate buffer (pH 7.2) containing 0.1% CaCl_2_. The samples were imbedded in an Epon 812 mixture and polymerized in an oven at 60 °C for 36 h. An Ultracut UC7 ultramicrotome (Leica, Austria) was used to cut and mount ultrathin sections (70 nm thickness) on 75-mesh copper grids. After uranyl acetate counterstaining for 10 min and lead citrate staining for 7 min, the sections were viewed using a KBSI Bio-High Voltage Electron Microscope (JEOL Ltd., Tokyo, Japan).

### Extracellular acidification rate (ECAR) analyses

The ECAR was measured using a Seahorse Bioscience XF96 Analyzer (Agilent Technologies). A total of 3 × 10^4^ phorbol 12-myristate 13-acetate (PMA)-differentiated THP-1 cells were seeded in an XF96 plate and incubated overnight. The ECAR was measured using a Seahorse XF Glycolysis Stress Test Kit (Agilent Technologies). The process is described in detail in the Supplementary Information.

### Statistical analysis

The data were analyzed using Prism version 5 or 10 (GraphPad). *P* values were calculated via one-way analysis of variance (ANOVA) with Tukey’s or Bonferroni’s multiple comparison test or an unpaired *t*-test with the Mann‒Whitney test.

Other methods are described in detail in the Supplementary Information.

## Results

### A30L upregulates the inflammatory response in human immune cells

**Fig. 1:**
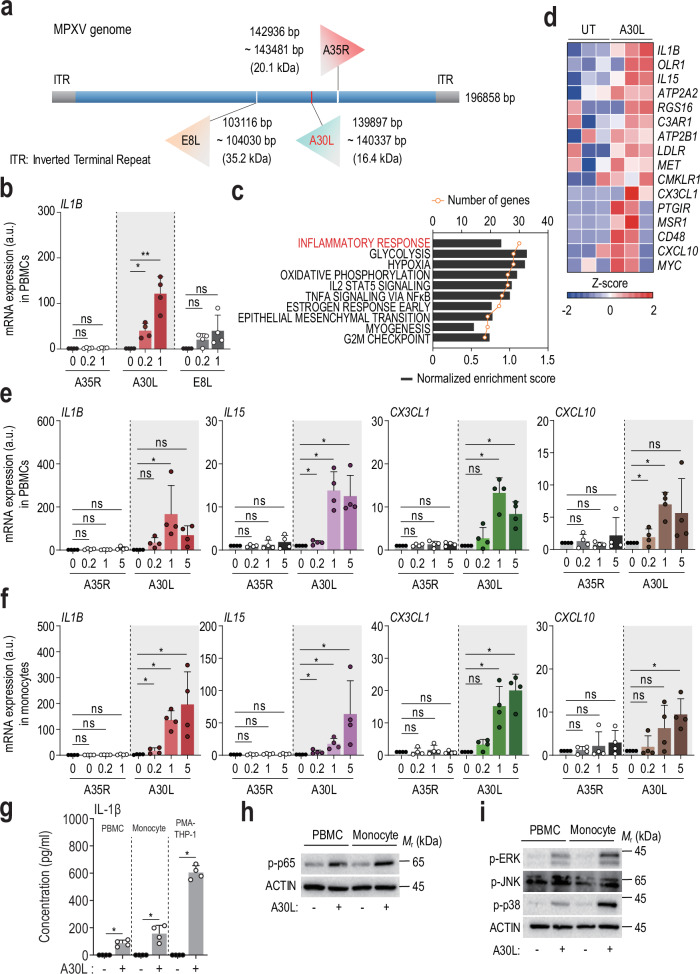
The A30L protein enhances the inflammatory response in human PBMCs and monocytes. **a** Schematic overview and domain organization of the MPXV E8L, A35R, and A30L proteins used in this study. **b** PBMCs were treated with A35R, A30L, or E8L (0.2 or 1 μg/mL) for 6 h. Total RNA extracted from the cells was then subjected to qRT-PCR analysis to measure the *IL1B* mRNA expression level. **c** Gene Ontology analysis of genes whose expression was upregulated in A30L-treated PBMCs. The list was ranked by the number of overlapping genes. **d** Heatmap showing the expression of inflammatory response-related genes in untreated and A30L-treated human PBMC samples. *IL1Β*, *IL15*, *CX3CL1*, and *CXCL10* mRNA expression levels in human PBMCs (**e**) or monocytes (**f**) treated with A35R or A30L (0.2, 1, or 5 μg/mL for 6 h). **g** IL-1β concentrations in the supernatants of the indicated cells (human PBMCs, human monocytes, PMA-differentiated THP-1 cells, and murine BMDMs) treated with A30L (5 μg/ml for 18 h) were measured via ELISA. Western blots showing the levels of phosphorylated NF-κB (p-p65) (**h**), phosphorylated ERK (p-ERK), phosphorylated JNK (p-JNK), and phosphorylated p38 (p-p38) (**i**) in A30L-treated PBMCs or monocytes (5 μg/ml for 18 h). Statistical analysis was performed via one-way ANOVA with Tukey’s multiple comparison test or an unpaired *t*-test with the Mann‒Whitney test, and the results are presented as the means ± SDs. *p < 0.05; **p < 0.01. a.u. arbitrary unit, ns not significant.

A study of viral genes/proteins affecting host immune responses provided insights into the immunomodulatory abilities of poxvirus proteins^6^. However, the poxvirus proteins involved in distinct host immunopathogenic responses during infection are unknown. We assessed the inflammatory activities of the A35R, A30L, and E8L MPXV envelope proteins by measuring the *IL1B* mRNA level in human PBMCs. A30L, a 146-amino-acid envelope protein of unknown function of the MPXV^29^ (Fig. 1a), strongly induced *IL1Β* expression in human PBMCs (Fig. 1b). Next, we performed an RNA-seq analysis to evaluate the A30L-induced transcriptomic profile. Notably, A30L triggered the expression of gene clusters associated with inflammation (Fig. 1c). The heatmap analysis indicated that A30L strongly stimulated the expression of *IL1Β*, *IL15*, *CX3CL1*, and *CXCL10* in human PBMCs (Fig. 1d). Quantitative real-time polymerase chain reaction (qRT-PCR) confirmed that the expression levels of *IL1Β*, *IL15*, *CX3CL1*, and *CXCL10* were significantly increased by A30L but not by A35R in human PBMCs and monocytes (Fig. 1e, f). Moreover, treatment with A30L (5 μg/mL) significantly increased the secretion of IL-1β from human primary PBMCs, monocytes, and PMA-differentiated THP-1 cells (Fig. 1g).

We examined the A30L-induced activation of the NF-κB and mitogen-activated protein kinase (MAPK) signaling pathways by western blotting to investigate the molecular mechanisms by which A30L induces IL-1β secretion. The phosphorylation levels of NF-κB (p65) and MAPKs (ERK1/2, JNK1/2, and p38 MAPK) were increased in A30L-treated human primary PBMCs and monocytes (Fig. 1h, i). Taken together, these results suggest that A30L activates inflammatory signaling, as well as the production of cytokines and chemokines, in human primary PBMCs and monocytes.

### A30L triggers the activation of the NLRP3 inflammasome in human immune cells

**Fig. 2:**
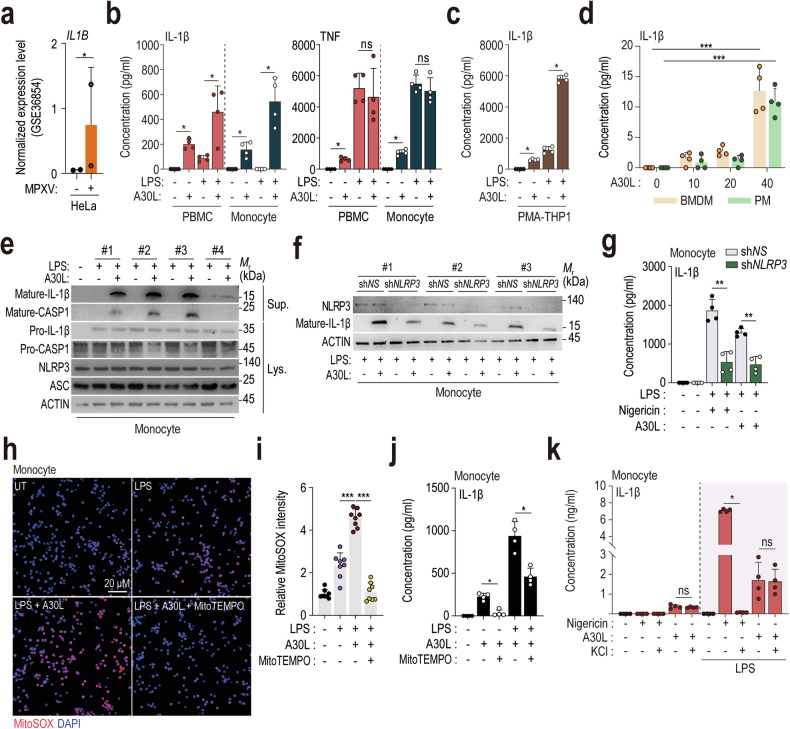
A30L activates the NLRP3 inflammasome in human monocytes. **a** Boxplot of *IL1B* expression levels in uninfected and MPXV-infected HeLa cells (from the GSE36854 cohort). **b** IL-1β and TNF concentrations in the supernatants of the indicated cells (human PBMCs and monocytes) stimulated with LPS (10 ng/mL for 4 h) followed by A30L (5 μg/mL for 18 h) were measured via ELISA. **c** IL-1β concentrations in the supernatants of PMA-differentiated THP-1 cells stimulated with LPS (10 ng/mL for 4 h) followed by A30L (5 μg/mL for 18 h), as measured by ELISA. **d** IL-1β concentrations in the supernatants of murine BMDMs and PMs stimulated with A30L (with the indicated concentration for 18 h), as measured by ELISA. **e** Western blot analysis of mature IL-1β and caspase-1 levels in culture supernatants (Sup.) and pro-IL-1β, pro-caspase-1, NLRP3, and ASC levels in cell lysates (Lys.) from human primary monocytes treated with A30L (5 μg/mL for 18 h) after LPS priming. **f** Western blot analysis of NLRP3 and mature IL-1β levels in human primary monocytes treated with A30L (5 μg/mL for 18 h) after NLRP3 knockdown and LPS priming. **g** IL-1β concentrations in the supernatants of monocytes stimulated with LPS (10 ng/mL for 4 h) followed by A30L (5 μg/mL for 18 h) after NLRP3 knockdown, as measured by ELISA. Representative images of immunofluorescence staining (scale bar = 20 μm; **h**) and quantitative analysis of the relative fluorescence intensities of DAPI and mtROS (**i**) in A30L-treated human primary monocytes (5 μg/mL for 4 h) after pretreatment with MitoTEMPO (100 μM for 2 h). The mtROS and nuclei were stained with MitoSOX Red (red) and DAPI (blue), respectively. IL-1β concentrations in the culture supernatants of cells incubated in the presence or absence of MitoTEMPO (**j**) or KCl (**k**). Statistical analysis was performed with an unpaired *t*-test with the Mann‒Whitney test or one-way ANOVA with Tukey’s multiple comparison test, and the results are presented as the means ± SDs. *p < 0.05; **p < 0.01; ***p < 0.001. ns not significant.

We analyzed the expression of *IL1Β* using publicly available transcriptomic datasets (GSE21001 and GSE36854) derived from MPXV-infected human epithelial HeLa cells and rhesus monkey kidney epithelial MK2 cells to investigate the potential role of IL-1β in mpox immunopathogenesis^30^. The expression of *IL1Β* was elevated in MPXV-infected HeLa cells compared with that in uninfected controls (Fig. 2a), suggesting that increased *IL1B* expression is related to mpox in humans. We then measured the secretion of IL-1β and TNF-α from human primary PBMCs and monocytes in the presence or absence of LPS priming. At 5 μg/mL, A30L significantly increased the secretion of IL-1β, but not TNF, from LPS-primed PBMCs and human primary monocytes (Fig. 2b). Additionally, stimulation with 5 μg/mL A30L significantly upregulated IL-1β expression in PMA-differentiated THP-1 cells (Fig. 2c). Furthermore, 40 μg/mL A30L significantly increased IL-1β production in murine BMDMs and PMs (Fig. 2d). These results demonstrate that A30L triggers an inflammatory response in both human immune cells and murine macrophages, with human cells exhibiting a stronger inflammatory response than murine macrophages.

Upon assembly, the NLRP3 inflammasome activates caspase-1, leading to the release of IL-1β and IL-18. Aberrant secretion of NLRP3 inflammasome-related cytokines is implicated in the pathogenesis of several inflammatory disorders^9,10^. We treated LPS-primed human primary monocytes with A30L to evaluate the effects of A30L on IL-1β secretion and NLRP3 inflammasome activation in human immune cells, and detected significant cleavage of pro-caspase-1 and maturation of IL-1β (Fig. 2e).

We investigated the role of NLRP3 in A30L-induced activation of the inflammasome complex by employing a short hairpin RNA (shRNA) targeting *NLRP3* (sh*NLRP3*) to knock down its expression in human primary monocytes. The suppression of NLRP3 expression via sh*NLRP3* led to a significant reduction in A30L-induced IL-1β secretion and maturation in human monocytes (Fig. 2f, g) compared with those in cells transduced with a nonspecific shRNA (sh*NS*). We further explored the mechanisms by which A30L activates the NLRP3 inflammasome. A30L treatment significantly increased mitochondrial reactive oxygen species (mtROS) generation in LPS-primed human monocytes (Fig. 2h, i). The mtROS scavenger MitoTEMPO effectively inhibited A30L-induced IL-1β secretion from both LPS-primed monocytes (Fig. 2j) and murine BMDMs (Supplementary Fig. 1). In contrast, extracellular K^+^ (5 mM) did not impact A30L-induced IL-1β secretion (Fig. 2k). These findings indicate that A30L-induced activation of the NLRP3 inflammasome is mediated by mtROS generation.

### IAMP29, a core peptide of A30L, induces inflammatory responses in human immune cells

**Fig. 3:**
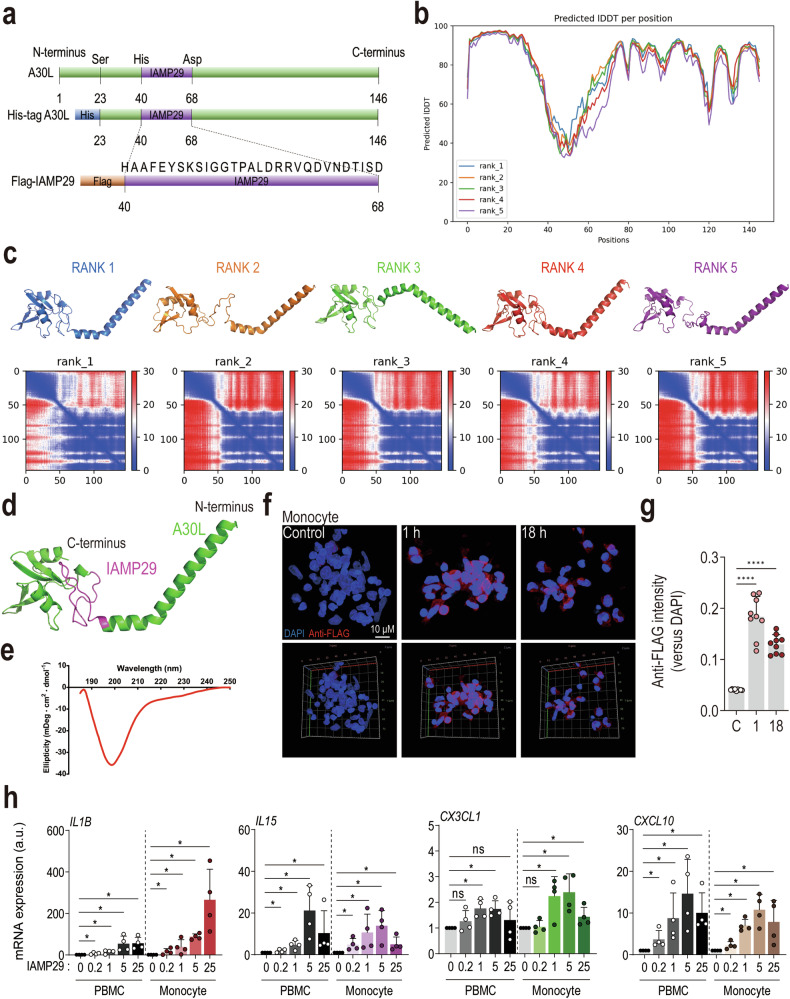
IAMP29, a core peptide of A30L, induces inflammatory responses in human monocytes. **a** Schematic overview and sequence of the studied IAMP29 peptide. **b** Models of the top 5 ranked prediction results, featuring predicted aligned error diagrams showing two distinct domains: the N-terminal helical region and the C-terminal core region. **c** Predicted IDDT per position, indicating the confidence of the model at each residue. All five models exhibited similar values. **d** Structural prediction of the MPXV A30L protein using AlphaFold2. **e** CD spectrum of the IAMP29 peptide, displaying a typical curve of a random coil. These findings indicate that residues 40–69 of the MPXV A30L protein are mostly unstructured in solution, which is consistent with the AlphaFold2 model. Representative images of immunofluorescence staining (scale bar = 10 μm) (**f**) and quantitative analysis of the relative fluorescence intensities of DAPI and anti-FLAG staining (**g**). Human primary monocytes were treated with IAMP29 (25 μg/mL for 1 or 18 h). FLAG-IAMP29 and nuclei were stained with an anti-FLAG antibody (red) and DAPI (blue), respectively. **h** *IL1Β*, *IL15*, *CX3CL1*, and *CXCL10* mRNA expression levels in IAMP29-treated human PBMCs or monocytes. Statistical analysis was conducted with an unpaired *t*-test with the Mann‒Whitney test or one-way ANOVA with Tukey’s multiple comparison test, and the results are presented as the means ± SDs. *p < 0.05; ****p < 0.0001. a.u. arbitrary unit, ns not significant.

We used the primary sequence of recombinant A30L (124 amino acids [Ser23 to Leu146]; Fig. 3a) to search several databases and identify the region of MPXV A30L responsible for NLRP3 inflammasome activation. Intriguingly, one region displayed partial structural similarity with the Golgi dynamics (GOLD) domain based on the SWISS-MODEL prediction (https://swissmodel.expasy.org/). GOLD domain proteins include the p24(p24/gp25L/emp24/Erp) family, the members of which are present in coated vesicles that transport cargo from the endoplasmic reticulum (ER) to the Golgi complex^31^. The GOLD domain-like core peptide of MPXV A30L was named IAMP29 (inflammasome-activating peptide consisting of 29 amino acids [His40 to Asp68]; Fig. 3a). IAMP29 of MPXV A30L was predicted using AlphaFold2 (Fig. 3b, c). IAMP29 is located in a flexible region of A30L that connects the long N-terminal helical segment and compact C-terminal domain (Fig. 3d). The region is highly dynamic, according to CD spectroscopy using the synthetic peptide (Fig. 3e).

We next investigated the function of IAMP29. We used confocal microscopy to determine whether IAMP29 is internalized and present within human cells, and the results revealed that IAMP29 was localized in the cytosol of human monocytes for up to 18 h (Fig. 3f, g). Flow cytometry further confirmed the internalization of FITC-labeled IAMP29 in human primary monocytes (Supplementary Fig. 2a, b). We then examined the ability of IAMP29 to induce the expression of *IL1Β*, *IL15*, *CX3CL1*, and *CXCL10* in human PBMCs. Like A30L, IAMP29 robustly induced the expression of these inflammatory cytokines and chemokines (Fig. 3h). Collectively, these data indicate that the core peptide IAMP29 of A30L penetrates immune cells and activates inflammatory responses.

### IAMP29-driven NLRP3 inflammasome activation depends on mtROS generation in monocytes and macrophages

**Fig. 4:**
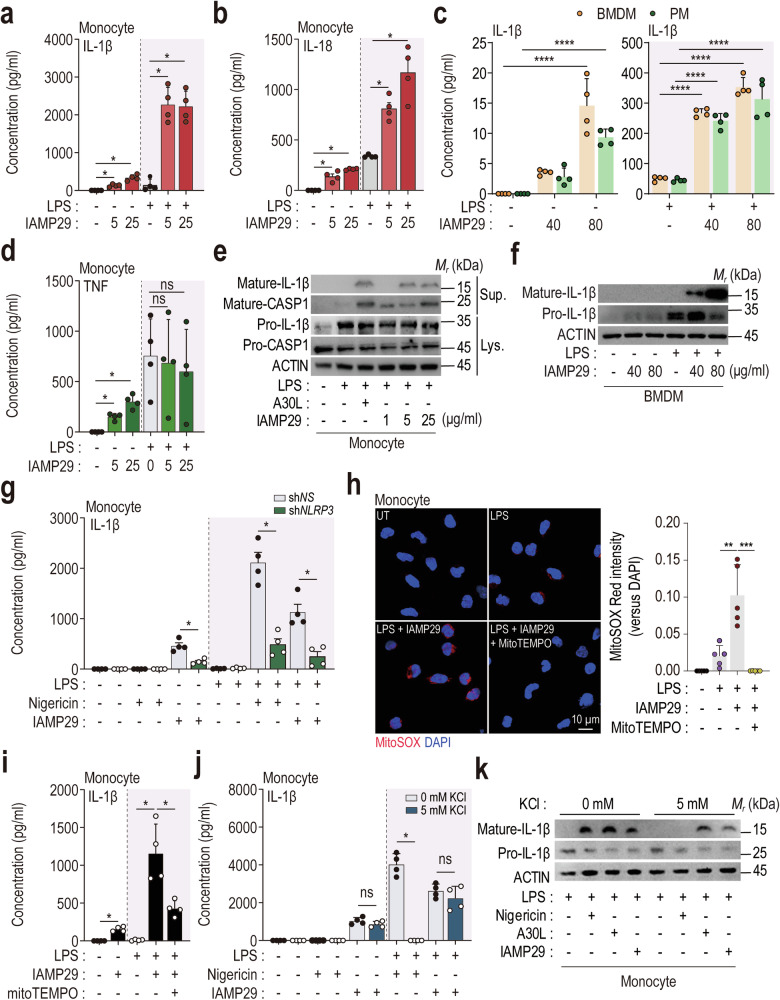
IAMP29 activates the NLRP3 inflammasome in human monocytes and murine BMDMs. Human IL-1β (**a**), human IL-18 (**b**), murine IL-1β (**c**), and human TNF (**d**) concentrations in the supernatants of the indicated IAMP29-treated cells after LPS priming, as measured by ELISA. **e** Western blots showing the levels of mature IL-1β and mature caspase-1 in culture supernatants (Sup.) and pro-IL-1β, pro-caspase-1, and β-actin in cell lysates (Lys.) from IAMP29-treated human primary monocytes (1, 5, or 25 μg/mL for 18 h) after LPS priming. **f** Western blots showing the levels of mature IL-1β in culture supernatants and pro-IL-1β and β-actin in lysates from IAMP29-treated murine BMDMs (40 or 80 μg/mL for 18 h) after LPS priming. **g** IL-1β concentrations in the supernatants of NLRP3-knockdown monocytes treated with IAMP29 (5 μg/mL for 18 h) after LPS priming, as measured with ELISA. **h** Representative images of immunofluorescence staining (scale bar = 10 μm) and quantitative analysis of the relative fluorescence intensities of DAPI and mtROS. Human primary monocytes were treated with IAMP29 (25 μg/mL for 4 h) after pretreatment with MitoTEMPO (100 μM for 2 h). The mtROS and nuclei were stained with MitoSOX Red (red) and DAPI (blue), respectively. IL-1β concentrations in the culture supernatants of cells incubated in the presence or absence of MitoTEMPO (**i**) or KCl (**j**). **k** Western blots showing the levels of mature IL-1β in culture supernatants and pro-IL-1β in the lysates of LPS-primed human primary monocytes treated with IAMP29 (5 μg/mL for 18 h) after KCl pretreatment (5 mM for 2 h). Statistical analysis was conducted with an unpaired *t*-test with the Mann‒Whitney test or one-way ANOVA with Tukey’s multiple comparison test, and the results are presented as the means ± SDs. *p < 0.05; **p < 0.01; ***p < 0.001; ****p < 0.0001. ns not significant.

Because A30L induced NLRP3 inflammasome activation in human monocytes, we investigated whether IAMP29 induced the secretion of IL-1β and IL-18 by human primary monocytes. Human primary monocytes were primed with LPS (10 ng/mL) for 4 h, followed by an incubation with IAMP29 (5 and 25 μg/mL) for 24 h. The application of 1–25 μg/mL IAMP29 to LPS-primed human monocytes significantly increased the secretion of IL-1β and IL-18 (Fig. 4a, b). The application of 20–80 μg/mL IAMP29 to murine BMDMs and PMs significantly increased IL-1β production (Fig. 4c). Notably, no discernible increase in TNF-α production was observed in LPS-primed human monocytes in response to IAMP29 treatment (Fig. 4d). Furthermore, the maturation of IL-1β was significantly increased in human primary monocytes and murine BMDMs after treatment with LPS or IAMP29 compared with the control cells (Fig. 4e, f). Additionally, knocking down NLRP3 using sh*NLRP3* substantially downregulated IAMP29-mediated IL-1β production in human monocytes (Fig. 4g), confirming that NLRP3 inflammasome activation by IAMP29 is dependent on NLRP3.

We next explored whether the activation of the NLRP3 inflammasome by IAMP29 depends on increased mtROS generation. Compared with the control and LPS-only treatments, MitoSOX staining revealed significant increases in mtROS levels after treatment with LPS and IAMP29 (Fig. 4h, human primary monocytes; Supplementary Fig. 3a, murine BMDMs). Moreover, after treatment with MitoTEMPO, LPS/IAMP29-induced mtROS generation was significantly reduced compared with that in the control and LPS-only groups (Fig. 4h). Consistent with these findings, the increased secretion of IL-1β induced by LPS/IAMP29 was significantly reduced by MitoTEMPO (Fig. 4i and Supplementary Fig. 3b). Interestingly, the secretion of mature IL-1β induced by IAMP29 was not influenced by K^+^ efflux; maintaining a high extracellular K^+^ concentration did not affect the production or maturation of IL-1β in human primary monocytes (Fig. 4j, k). These results suggest that IAMP29 plays a substantial role in activating the NLRP3 inflammasome through mtROS generation in monocytes and macrophages.

### IAMP29 interacts with PKM, which is required for NLRP3 inflammasome activation

**Fig. 5:**
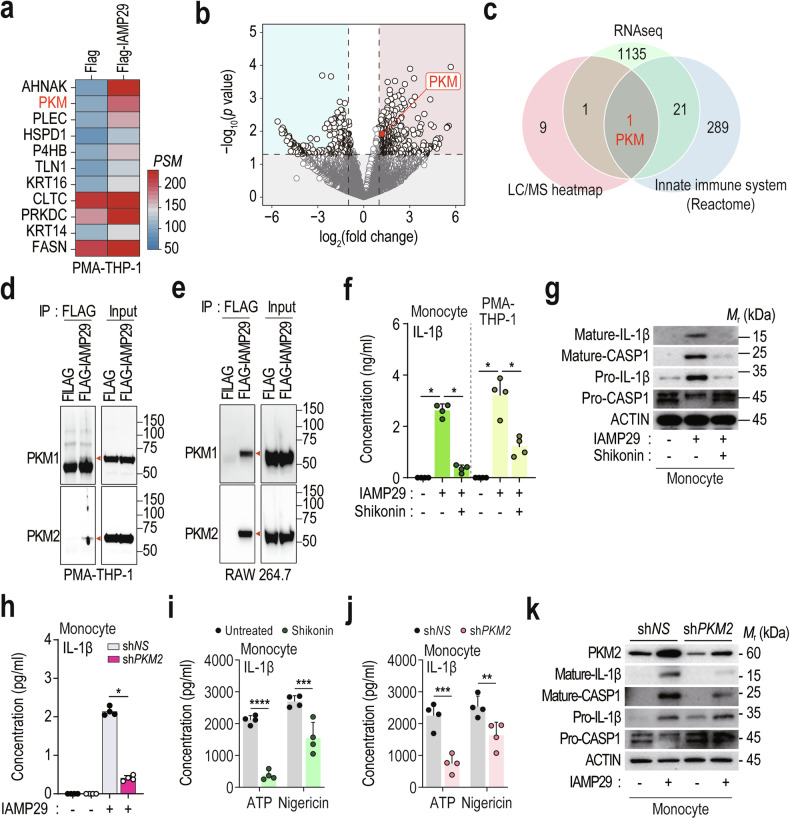
IAMP29 activates the NLRP3 inflammasome via an interaction with PKM2. **a** Heatmap showing the top protein hits identified by the LC‒MS analysis in PMA-differentiated THP-1 cells with FLAG or FLAG-IAMP29. **b** Volcano plot depicting the significant increase in *PKM* gene expression in A30L-treated human primary PBMCs. **c** Venn diagram illustrating the overlap of PKM2 in the three indicated groups. **d**, **e** Validation using PKM1 and PKM2 antibodies to detect immunoprecipitates from FLAG- or FLAG-IAMP29-treated cells (PMA-differentiated THP-1 and RAW 264.7 cells). **f** IL-1β concentrations in the supernatants of the indicated cells treated with IAMP29 (25 μg/mL for 18 h) after pretreatment with shikonin (5 μM for 2 h), as measured by ELISA. **g** Western blots showing the levels of mature IL-1β and mature caspase-1 in culture supernatants and pro-IL-1β, caspase-1, and β-actin in lysates of IAMP29-treated monocytes cultured in the absence or presence of shikonin (5 μM for 2 h). **h** IL-1β concentrations in the supernatants of IAMP29-treated monocytes (25 μg/mL for 18 h) with PKM2 knockdown, as measured by ELISA. IL-1β concentrations in the supernatants of shikonin-treated (**i**) or *PKM2*-knockdown monocytes (**j**) treated with the indicated NLRP3 inflammasome activators were measured by ELISA. **k** Western blots showing the levels of mature IL-1β and mature caspase-1 in culture supernatants and PKM2, pro-IL-1β, pro-caspase-1, and β-actin in the lysates of IAMP29-treated monocytes after PKM2 knockdown. Statistical analysis was conducted with an unpaired *t*-test with the Mann‒Whitney test or one-way ANOVA with Tukey’s multiple comparison test, and the results are presented as the means ± SDs. *p < 0.05; **p < 0.01; ***p < 0.001; ****p < 0.0001.

PMA-differentiated THP-1 cells were treated with a FLAG-tagged IAMP29 peptide to identify the mechanism(s) underlying IAMP29-induced NLRP3 inflammasome activation. IP was subsequently performed using anti-Flag® M2 affinity gel, and the IAMP29 immunocomplexes were subjected to mass spectrometry to identify the protein(s) responsible for NLRP3 inflammasome activation (Supplementary Table 2). The heatmap analysis revealed 11 significantly differentially bound proteins, filtered with a fold change > 1.5 compared with the control (anti-FLAG), and a peptide-spectrum match (PSM) of anti-FLAG-IAMP29 > 100 (Fig. 5a). Furthermore, the proteins overlapped with the significantly upregulated genes in A30L-treated human PBMCs (RNA-seq; Supplementary Table 3) and with 301 innate immune system-related proteins (Reactome database) (Fig. 5b, c). A PKM protein included in all three groups was considered a candidate interactor with IAMP29. In the last step of glycolysis, PKM transfers a phosphate group from phosphoenolpyruvate to adenosine diphosphate (ADP), forming pyruvate and ATP^32^. Notably, PKM2-mediated glycolysis promotes inflammasome activation by modulating protein kinase R (PKR) phosphorylation in macrophages^32^. Consequently, we investigated PKM2 as a mediator of IAMP29-induced NLRP3 inflammasome activation.

We conducted IP followed by western blotting to examine the physical interaction between the IAMP29 and PKM1/2 proteins and validate the aforementioned findings. Our results indicated that FLAG-tagged IAMP29 interacted with endogenous PKM1 and PKM2 in PMA-differentiated THP-1 monocytes (Fig. 5d) and RAW 264.7 cells (Fig. 5e). We employed shikonin, a pharmacological inhibitor of PKM2^33^, to further explore the role of PKM2 in IAMP29-induced inflammasome activation. ELISAs revealed that shikonin inhibited IL-1β release from primed monocytes (Fig. 5f). Moreover, Western blot analysis demonstrated that shikonin reduced the levels of IL-1β and cleaved caspase-1 in the culture supernatants of human monocytes stimulated with IAMP29 (Fig. 5g).

We suppressed PKM2 expression by transducing cells with a lentivirus carrying PKM2-specific shRNA (sh*PKM2*) to further corroborate these findings. Knockdown of PKM2 via sh*PKM2* significantly reduced IL-1β release from LPS-primed human monocytes stimulated with IAMP29 (Fig. 5h). Both shikonin treatment and PKM2 silencing significantly diminished the IL-1β secretion triggered by classical NLRP3 activators, such as ATP and nigericin (Fig. 5i, j). Additionally, the knockdown of PKM2 decreased the levels of mature IL-1β and cleaved caspase-1 in the culture supernatants of LPS-primed monocytes stimulated with IAMP29 (Fig. 5k). These findings strongly suggest that IAMP29 interacts with PKM2, thereby activating the NLRP3 inflammasome in human monocytes and murine macrophages.

### IAMP29 triggers the transcriptional activation of PKM2 and activates the NLRP3 inflammasome via PKR signaling

**Fig. 6:**
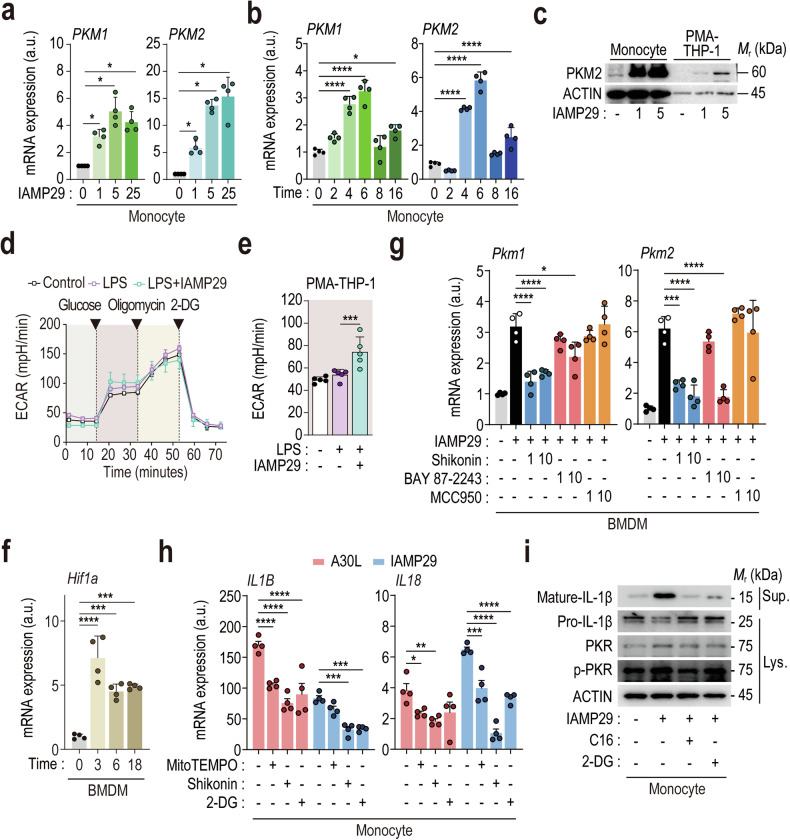
IAMP29 treatment increases the PKM2 mRNA expression level and activates the NLRP3 inflammasome via PKR signaling. Dose (**a**)- or time (**b**)-dependent expression of *PKM1* and *PKM2* in IAMP29-treated human monocytes. **c** Western blots showing the levels of PKM2 in the lysates of IAMP29-treated human monocytes or PMA-differentiated THP-1 cells. **d**, **e** The ECAR in PMA-differentiated THP-1 cells treated with IAMP29 (25 μg/mL for 18 h) after LPS stimulation. **f** *Hif1a* mRNA expression in IAMP29-treated murine BMDMs (25 μg/mL for 3, 6, and 18 h). **g** *Pkm1* and *Pkm2* mRNA expression in IAMP29-treated murine BMDMs after pretreatment with the indicated inhibitors. **h** *IL1B* and *IL18* mRNA expression in A30L- or IAMP29-treated human monocytes after pretreatment with the indicated inhibitors. **i** Western blots showing the levels of mature IL-1β in culture supernatants and pro-IL-1β, PKR, and phospho-PKR in the lysates of IAMP29-treated monocytes cultured in the presence of the indicated inhibitors. Statistical analysis was conducted with an unpaired *t-*test with the Mann‒Whitney test or one-way ANOVA with Tukey’s multiple comparison test, and the results are presented as the means ± SDs. *p < 0.05; **p < 0.01; ***p < 0.001; ****p < 0.0001. a.u. arbitrary unit, ns not significant.

We next investigated the kinetics of the *PKM1* and *PKM2* mRNAs, as well as glycolysis, in human monocytes and murine BMDMs in response to IAMP29. Both A30L and IAMP29 increased the *PKM1* and *PKM2* mRNA expression levels in human primary monocytes (Fig. 6a and Supplementary Fig. 4a). In IAMP29-treated murine BMDMs, the *Pkm1* and *Pkm2* mRNA expression levels were also increased in a dose-dependent manner (Supplementary Fig. 4b). Both cell types presented early peaks (3–6 h) in *PKM1* and *PKM2* mRNA expression after stimulation with IAMP29 (Fig. 6b and Supplementary Fig. 4c). Notably, compared with human monocytes, BMDMs presented a smaller increase in *Pkm1* and *Pkm2* mRNA levels, even after treatment with higher IAMP29 doses (20–80 μg/mL) (Fig. 6a and Supplementary Fig. 4b). IAMP29 treatment also increased PKM2 protein levels in human monocytes and PMA-differentiated THP-1 cells (Fig. 6c). We measured the ECAR after the sequential addition of glucose (10 mM), oligomycin (1 mM), and 2-deoxy-glucose (2-DG; 50 mM) to assess glycolysis (Fig. 6d and Supplementary Fig. 5a). Glycolysis was significantly increased in PMA-differentiated THP-1 cells (Fig. 6e) and RAW 264.7 cells (Supplementary Fig. 5b) treated with IAMP29 for 18 h compared with those treated with LPS or the control. Because hypoxia-inducible factor (HIF)-1α acts as a transcription factor that promotes glycolysis and activates PKM^34^, we treated murine BMDMs with IAMP29 and observed a significant increase in *Hif1a* mRNA levels (Fig. 6f). Furthermore, inhibition of HIF-1α with BAY 87-2243 significantly reduced the expression of both *PKM1* and *PKM2* (Fig. 6g), indicating that IAMP29-induced PKM1/2 expression depends on HIF-1α.

We assessed whether IAMP29-induced pro-IL-1β and pro-IL-18 expression is mediated by mtROS, PKM2, and glycolysis to examine how IAMP29-induced PKM2 activation facilitates NLRP3 inflammasome activation. In human monocytes and murine BMDMs, PKM2 (shikonin) and glycolysis (2-DG) inhibitors significantly inhibited IAMP29-triggered pro-IL-1β and pro-IL-18 mRNA expression (Fig. 6h and Supplementary Fig. 5c), suggesting that PKM2 and glycolysis are crucial for Signal 1 activation by IAMP29. Additionally, PKM2 stimulates PKR phosphorylation, activating various inflammasomes in macrophages^32,35^. Notably, IAMP29 robustly induced PKR phosphorylation and IL-1β maturation, which was reduced by the PKR inhibitor C16^36^, in LPS-primed monocytes (Fig. 6i). These findings indicate that IAMP29-induced PKM2 transcription is crucial for NLRP3 inflammasome activation via mechanisms such as mtROS production, enhanced glycolysis, and PKR pathway activation.

### IAMP29 restricts the intracellular growth of NTMs in macrophages

**Fig. 7:**
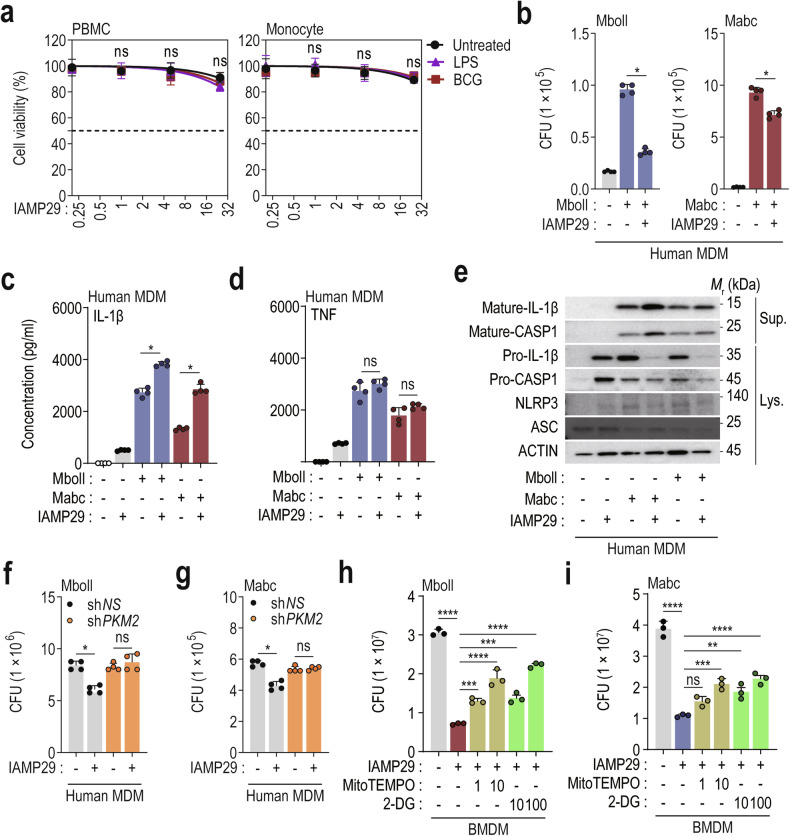
IAMP29 treatment restricts the intracellular growth of NTMs in human and murine macrophages. **a** The cytotoxicity of IAMP29 in PBMCs and monocytes was assessed via a CCK-8 assay. The cells were treated with IAMP29 (0.2, 1, 5, or 25 μg/mL for 24 h) after LPS or BCG pretreatment. **b** Intracellular survival of smooth strains of Mboll and Mabc in IAMP29-treated human primary MDMs after Mboll or Mabc infection (MOI = 1 for 2 h), as determined by a CFU assay. IL-1β (**c**) and TNF (**d**) concentrations in the supernatants of IAMP29-treated human MDMs. The cells were treated with IAMP29 (25 μg/mL for 18 h) after Mboll or Mabc infection (MOI = 1 for 2 h). **e** Western blots showing the levels of mature IL-1β and mature caspase-1 in culture supernatants (Sup.) and pro-IL-1β, pro-caspase-1, NLRP3, and ASC in cell lysates (Lys.) of Mboll- or Mabc-infected human MDMs treated with IAMP29 (25 μg/mL for 18 h). Intracellular survival of Mboll (**f**) and Mabc (**g**) in human MDMs with PKM2 knockdown after Mboll or Mabc infection (MOI = 1 for 2 h), as determined by a CFU assay. Intracellular survival of Mboll (**h**) and Mabc (**i**) in Mboll- or Mabc-infected murine BMDMs treated with IAMP29 (40 μg/mL for 18 h) after pretreatment with MitoTEMPO (1 and 10 μM for 2 h) or 2-DG (10 and 100 μM for 2 h), as determined by a CFU assay. Statistical analysis was conducted with an unpaired *t*-test with the Mann‒Whitney test or one-way ANOVA with Tukey’s multiple comparison test, and the results are presented as the means ± SDs. *p < 0.05; **p < 0.01; ***p < 0.001; ****p < 0.0001. ns not significant.

Activation of the NLRP3 inflammasome is essential for host defense against Mabc, a rapidly growing NTM with high antibiotic resistance^20^. We examined the cytotoxicity of IAMP29 in PBMCs and monocytes from healthy volunteers. The application of 0.2–25 μg/mL IAMP29 for 24 h did not exert significant cytotoxic effects on human primary PBMCs or monocytes (Fig. 7a). Moreover, LPS or *M. bovis* Bacillus Calmette-Guérin (BCG) priming affected IAMP29 cytotoxicity (Fig. 7a). Consequently, we investigated the effect of IAMP29 on the antimicrobial response to infection with smooth strains of Mabc or Mboll in human MDMs. Human primary MDMs were infected with Mabc or Mboll (MOI of 1) and treated with IAMP29 (5 μg/mL). Next, they were subjected to an assessment of intracellular mycobacterial survival. IAMP29 at 5 μg/mL significantly suppressed the intracellular survival of Mabc or Mboll in human primary MDMs (Fig. 7b). IAMP29 significantly upregulated the production of IL-1β by Mabc- or Mboll-infected MDMs (Fig. 7c) but did not influence their secretion of TNF-α (Fig. 7d). Furthermore, IAMP29 activated caspase-1 and increased the level of active IL-1β in culture supernatants after Mabc or Mboll infection (Fig. 7e).

We next investigated the function of PKM2 in the antimicrobial responses triggered by IAMP29 in human MDMs. We introduced sh*PKM2* or control sh*NS* into MDMs, which were then induced to begin phagocytosis by Mabc or Mboll infection. The cells were cultured for 1 day and subjected to intracellular survival assays. Intracellular bacterial viability was significantly increased in human MDMs transduced with sh*PKM2*, whereas viability was decreased in sh*NS*-transduced cells (Fig. 7f, g). Additionally, both MitoTEMPO and 2-DG dose-dependently attenuated the IAMP29-induced antimicrobial responses (Fig. 7h, i). These findings highlight that IAMP29 activates antimicrobial responses in macrophages through PKM2, mtROS generation, and glycolysis during infections with Mabc and Mboll.

### IAMP29 treatment promotes leukemic cell death in vitro and in vivo

**Fig. 8:**
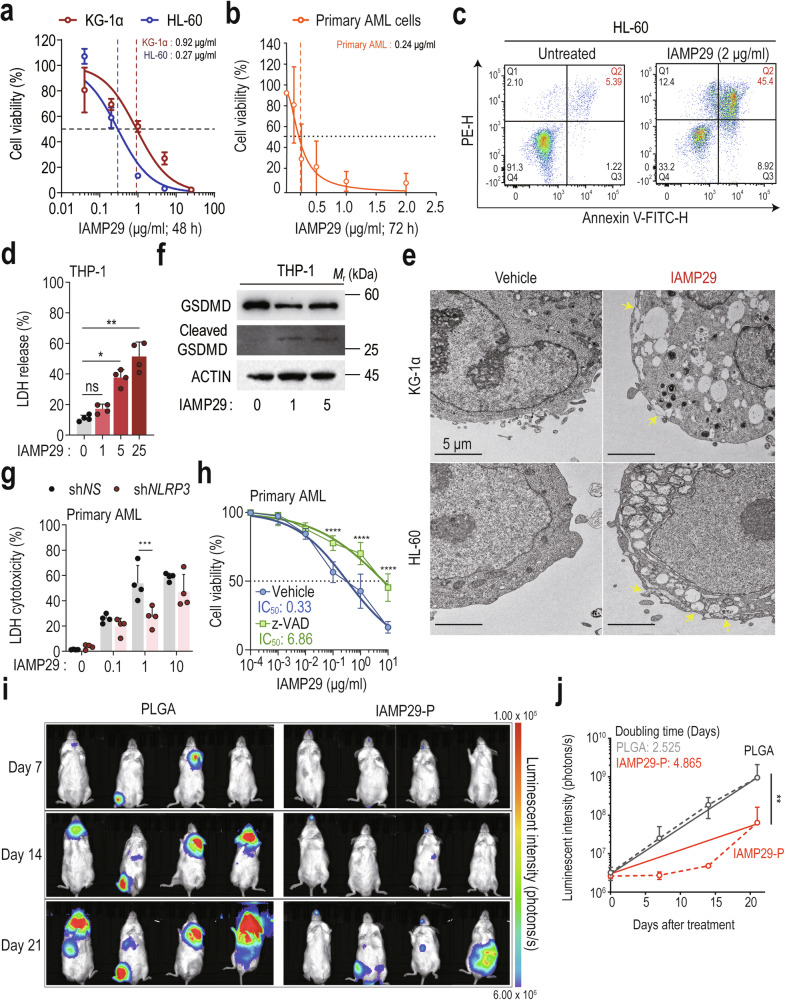
IAMP29 treatment activates apoptosis and pyroptosis in human leukemic cells. **a**, **b** The cytotoxic effect of IAMP29 on human KG-1α, HL-60 (48 h), and patient-derived AML cells (72 h) was assessed using a CCK-8 assay. **c** Flow cytometry analysis of IAMP29-treated HL-60 cells (2 μg/mL for 24 h) using annexin V/PI staining. **d** LDH release from IAMP29-treated THP-1 cells. The cells were treated with IAMP29 (1, 5, or 25 μg/mL for 24 h) and assessed with an LDH assay kit. **e** Transmission electron micrographs of HL-60 and KG-1α cells treated with IAMP29 (5 μg/mL for 24 h). **f** Western blots showing the levels of cleaved GSDMD in culture supernatants and GSDMD in the lysates of THP-1 cells treated with IAMP29 (1 and 5 μg/mL for 36 h). **g** LDH release from IAMP29-treated primary AML cells with NLRP3 knockdown. The cells were treated with IAMP29 (0.1, 1, or 10 μg/mL for 24 h) and assessed with an LDH assay kit. **h** The cytotoxic effect of IAMP29 in the presence or absence of z-VAD was assessed using a CCK-8 assay. **i** Luminescence intensity in NSG mice treated with PLGA or IAMP29-P (36 mg/kg) for 7 days after confirming the engraftment of K562-luc cells. NSG mice were orthotopically implanted with K562-luc cells (5 × 10^6^ cells/mouse) and injected with IAMP29-P through the tail vein twice a week for 2 weeks. The indicated dates represent “days” following the first IAMP29-P treatment. **j** Quantification of the luminescence intensity of photons released from each tumor in NSG mice treated with PLGA or IAMP29-P. Statistical analysis was conducted with an unpaired *t*-test with the Mann‒Whitney test or one-way ANOVA with Tukey’s multiple comparison test, and the results are presented as the means ± SDs. *p < 0.05; **p < 0.01; ***p < 0.001; ****p < 0.0001. ns not significant.

Several previous studies have shown that antimicrobial peptides also possess anticancer activity^37,38^. Pyroptosis enhances antitumor effects across various cancer types, including acute myeloid leukemia (AML), a highly heterogeneous malignancy with a poor prognosis that primarily affects older adults^39,40^. In this study, we evaluated the antileukemic potential of IAMP29 across multiple AML cell lines and primary AML cells. Treatment with IAMP29 at concentrations ranging from 0.04 to 25 µg/mL resulted in a drastic reduction in AML cell viability, with cytotoxicity exceeding 95% at 48 and 72 h. Notably, primary AML cells exhibited heightened sensitivity to lower concentrations, with an IC_50_ of 0.24 μg/mL, which was lower than that observed in various AML cell lines (Fig. 8a, b and Supplementary Fig. 6a–c).

Pyroptosis, which is characterized by the formation of membrane pores, increased LDH release, and an increased number of annexin V/PI-positive cells^41,42^, was assessed. The flow cytometry analysis indicated that IAMP29 increased the population of annexin V/PI-positive HL-60 cells, indicating late apoptosis and pyroptosis^41^ (Fig. 8c and Supplementary Fig. 6d). Additionally, IAMP29 treatment dose-dependently increased LDH release from THP-1 cells (Fig. 8d). Furthermore, IAMP29-treated HL-60 and KG-1α cells presented characteristic signs of pyroptosis, including cell swelling, a loss of plasma membrane integrity, and the disruption of intracellular organization with a decreased electron density, as observed by transmission electron microscopy^43^ (Fig. 8e). The terminal events of pyroptosis involve the activation of inflammatory caspases and the subsequent release of the N-terminal fragment of gasdermin D (GSDMD), leading to pore formation in the plasma membrane^44^. Notably, IAMP29 significantly increased the level of cleaved N-terminal GSDMD in THP-1 cells (Fig. 8f).

We knocked down NLRP3 in KG-1α cells, HL-60 cells, and primary AML cells and measured LDH release to confirm the induction of pyroptosis by IAMP29 in AML cells. Silencing NLRP3 markedly attenuated IAMP29-induced pyroptosis (Fig. 8g and Supplementary Fig. 6e, f) and reduced the cleaved N-terminal GSDMD level in HL-60 cells (Supplementary Fig. 6i). Importantly, NLRP3 knockdown did not completely abolish the anticancer effect of IAMP29. We thus explored whether IAMP29 induces apoptosis in AML cells. Pretreatment with z-VAD, a cell-permeant pancaspase inhibitor^45^, partially blocked cytotoxicity in primary AML cells (Fig. 8h), KG-1α cells (Supplementary Fig. 6g), and HL-60 cells (Supplementary Fig. 6h). These results strongly suggest that the antileukemic effects of IAMP29 depend on the activation of both apoptosis and pyroptosis.

We encapsulated IAMP29 in PLGA (IAMP29-P) polymeric nanoparticles^46^ to prevent peptidase degradation and to increase its therapeutic potential (Supplementary Fig. 7a). In a xenograft mouse model in which K562-luc cells expressing luciferase were implanted into NSG mice (*n* = 8 mice), treatment with IAMP29-P (36 mg/kg, twice weekly for 2 weeks) significantly slowed tumor progression compared with that in controls receiving only PLGA (Fig. 8i, j). Importantly, no differences in body weight were observed between the treatment and control groups at the study endpoint, indicating the safety of IAMP29-P treatment (Supplementary Fig. 7b). These findings highlight the potential of IAMP29 to suppress AML progression both in vitro and in vivo.

## Discussion

The roles of poxvirus proteins in modulating inflammasome activity and pyroptosis remain poorly understood. Our findings provide insights into the activation of the NLRP3 inflammasome by the MPXV envelope protein A30L and its core peptide IAMP29 in human monocytes and murine macrophages. We demonstrated that IAMP29 is internalized by human monocytes and induces NLRP3 inflammasome activation, enhancing both anti-NTM and anticancer responses. A recent structural modeling study revealed that several poxvirus proteins modulate inflammasome formation and pyroptosis^47,48^. For example, vaccinia A47L, a homolog of GSDMs, activates pyroptosis, whereas vaccinia C1L significantly enhances inflammasome activity^48^. Conversely, vaccinia F1L promotes viral virulence by interacting with NLRP1 and inhibiting inflammasome activation^47^. These studies, along with our data, highlight how poxviral effectors modulate host inflammasome activities, thereby influencing protective immune responses and viral pathogenesis. Therefore, further investigations are needed to determine the effects of poxvirus proteins on host innate immunity.

We identified a region of IAMP29 from the MPXV protein A30L that shows structural similarity to the GOLD domain. The GOLD domain is commonly associated with proteins such as p24 (p24/gp25L/emp24/Erp), which are found in coated vesicles and are responsible for transporting cargo from the ER to the Golgi complex^31^. The coiled-coil and GOLD domains of the p24 family protein, specifically transmembrane emp24 domain-containing protein 7 (TMED7), play a critical role in Toll-like receptor (TLR) 4 signaling by facilitating the trafficking of TLR4 from the ER to the cell surface^49^. Although the functions of GOLD domain-containing proteins in innate and inflammatory responses are not fully understood, our findings suggest that these structures are intricately involved in the regulation of innate immune signaling.

This study demonstrated that IAMP29 activates the inflammasome complex more robustly in human primary monocytes and monocytic cells than in murine macrophages. The underlying reason for this species-specific sensitivity to IAMP29 remains unclear; however, this finding suggests the potential affinity of the peptide of viral origin for targeting human immune cells more specifically. IAMP29 substantially induces mtROS generation in LPS-primed monocytes and monocytic cells, indicating that NLRP3 inflammasome activation is mediated by mitochondrial dysfunction. Additionally, IAMP29 drives immunometabolic remodeling toward aerobic glycolysis, culminating in the transcriptional activation of key genes involved in this pathway, such as *PKM1* and *PKM2*. Furthermore, the mass spectrometry analysis revealed binding interactions between IAMP29 and PKM1/PKM2, suggesting their roles in NLRP3 inflammasome activation. We also showed that IAMP29-induced inflammasome activation depends on multiple mechanisms, including mtROS generation, glycolysis, PKM2, and the PKR signaling pathway. Previous findings have shown that PKM2 can trigger the activation of the NLRP3 inflammasome and absent in melanoma 2 (AIM2) inflammasome by modulating PKR phosphorylation in macrophages^32^. Although we did not explore whether IAMP29 triggers AIM2 inflammasome activation, our findings implicate PKM1/2 in the assembly of the IAMP29-mediated inflammasome complex in human cells.

IAMP29 significantly enhanced intracellular antimicrobial responses to NTM, particularly smooth strains of Mabc and Mboll. Findings concerning the function of inflammasome activation during NTM infection are conflicting. Among individuals with lung diseases caused by members of the *M. avium* complex, the IL-1β response and NLRP3 inflammasome activation are decreased in PBMCs, monocytes, and macrophages compared with those in healthy controls, indicating increased host susceptibility^50^. Additionally, heterologous expression of the *esx*-1 region of *Mycobacterium marinum* in BCG induced AIM2 and NLRP3 inflammasome activity, leading to increased proportions of CD8^+^ T cells and polyfunctional CD4^+^ Th1 cells. These processes confer enhanced protection (relative to parental BCG) against highly virulent *M. tuberculosis*^51^. Furthermore, the secreted form of IL-1β, generated via caspase-1 activation, reduces intracellular *Mycobacterium kansasii* infection of human monocytes^52^. NLRP3 inflammasome activation is also required for the antimicrobial response to Mabc infection in human macrophages^20^. However, cytosolic oxidized mtDNA triggers IL-1β production during infection with rough Mabc, contributing to Mabc survival in murine macrophages^53^.

Our data indicate that IAMP29-induced antimicrobial responses rely on mtROS generation, PKM2 activation, and enhanced glycolysis. Studies have shown that mtROS play a crucial role in antimicrobial immune responses, although excessive mtROS production can be detrimental in certain pathologies^54^. Increased aerobic glycolysis and lactate levels have been found to increase the killing of intracellular *M. tuberculosis*. Notably, lactate significantly improves the clearance of *M. tuberculosis* in human macrophages through an autophagy-dependent mechanism^55^. IL-1 contributes to the host defense against *M. tuberculosis* by promoting eicosanoid production and inhibiting the excessive release of type I interferon^56^. Although we did not assess whether IAMP29-induced antimicrobial defenses involve eicosanoid production or a reduction in type I interferon secretion, our findings suggest that IAMP29 enhances anti-NTM defenses by activating inflammasome pathways through mtROS generation and promoting glycolysis.

A significant unmet need is effective medical therapies for relapsed/refractory or secondary AML, which is characterized by a poor prognosis and low response rates^57,58^. The NLRP3 inflammasome plays a dual role in cancer, impacting both cancer pathogenesis and therapeutic outcomes^39^. In this study, IAMP29 induced leukemia cell death by promoting two forms of programmed cell death—pyroptosis and apoptosis—while sparing normal monocytes and macrophages. Our data align with those of previous studies showing the antileukemic effects of pyroptosis-activating reagents. For example, small-molecule inhibitors targeting dipeptidyl dipeptidase (DPP) 8 and DPP9 trigger pyroptosis in human myeloid cells by interacting with the caspase recruitment domain (CARD)-containing protein CARD8^40^. In AML patients with TP53 mutations, who have a poor prognosis, DNA methyltransferase inhibitors activate endogenous retroviruses and trigger inflammasome signaling in a STING pathway-dependent manner^59^. However, few reports have described agents that can activate both pyroptosis and apoptosis for antileukemic effects. A recent study demonstrated that the knockdown of flotillin-1, a component and marker of lipid rafts, activated both apoptosis and pyroptosis, thereby suppressing AML cell growth^60^. Importantly, IAMP29 exerted antileukemic effects on primary cancer cells from AML patients. Therefore, IAMP29 shows promise as a therapeutic agent for hematopoietic cancers by activating both apoptosis and pyroptosis.

Several membranolytic and membrane-active peptides destabilize cellular homeostasis in microorganisms, functioning as both antimicrobial and anticancer agents^37,38^. Unlike these peptides, IAMP29 lacks structural features such as amphipathic structures and cationic amino acid residues, which facilitate interactions with multiple membranes^38^. Despite these findings, our data strongly indicate that IAMP29-induced NLRP3 inflammasome activation enables the peptide to generate both antimicrobial and anticancer activities. In summary, IAMP29 shows significant potential as an anti-infective and anticancer immunotherapeutic. These findings could pave the way for new innovations in the treatment of various cancers and infectious diseases.

## Supplementary information

Supplementary information

Supplementary Table 2

Supplementary Table 3

## Data Availability

The datasets used and/or analyzed during the current study are available from the corresponding author upon reasonable request.
