# Supplementary material for: The inflammasome-activating poxvirus peptide IAMP29 promotes antimicrobial and anticancer responses
Source: Exp Mol Med. 2024 Nov 1;56(11):2475–90. doi: 10.1038/s12276-024-01339-3 (PMC11612179; doi:10.1038/s12276-024-01339-3)
Supplement: Supplementary file 1 — Supplementary information [file 12276_2024_1339_MOESM1_ESM.pdf]

Supplementary information for

‘The inflammasome-activating poxvirus peptide IAMP29 promotes antimicrobial and anticancer responses’

Taylor Roh, Wonhyoung Seo, Minho Won, Woo Seok Yang, Asmita Sapkota, Eun-Jin Park, Sung-Ho Yun, Sang Min Jeon, Kyung Tae Kim, Bomi Lee, Gyoungah Ryu, Sang-Hee Lee, Jung-Min Shin, Hyo Jung Shin, Young Jae Kim, Young Lee, Chaeuk Chung, Ik-Chan Song, Hyun Kyu Song, Eun-Kyeong Jo

Eun-Kyeong Jo

Address: #232, Department of Microbiology, and Infection Control Convergence Research Center, Chungnam National University School of Medicine, Daejeon, 35015, Korea.

E-mail: hayoungj@cnu.ac.kr

Extended Methods

Materials

Nigericin (SML1779), phorbol-12-myristate-13-acetate (PMA; P8139), MitoTEMPO (SML0737), Imidazolo-oxindole PKR inhibitor C16 (I9785), 2-Deoxy-D-glucose (D8375), and dimethyl sulfoxide (DMSO) were purchased from Sigma-Aldrich (St. Louis, MO, USA). Adenosine triphosphate (ATP; R0441) were purchased from Thermo Scientific (Waltham, MA, USA). Lipopolysaccharide (LPS) was obtained from InvivoGen (San Diego, CA, USA). BAY 87-2243 (6980) was obtained from Tocris Bioscience (Bristol, UK). MCC950 (inh-mcc) was obtained from InvivoGen. Shikonin (HY-N0822) was purchased from MedChemExpress (Monmouth Junction, NJ, USA). MPXV A30L and E8L were obtained from ACRObiosystems (Newark, DE, USA), and MPXV A35R was purchased from Sino Biological (Beijing, China). Synthetic IAMP29 peptide was manufactured by Dandicure (Cheongju, South Korea). Anti-human IL-1β (1:1000, ab2105) antibody was purchased from Abcam (Cambridge, UK). Anti-β-actin (1:2000, #5125), anti-caspase-1 (1:1000, #2225), anti-PKM1 (1:1000, #7067), anti-PKM2 (1:1000, #4053), anti-gasdermin D (1:1000, #39754), anti-cleaved gasdermin D (1:1000; #36425), anti-rabbit IgG (1:2000, #7074), and anti-mouse IgG (1:2000, #7076) antibodies were obtained from Cell Signaling Technology (Danvers, MA, USA). Anti-NLRP3 (1:1000, AG-20B-0014) and anti-ASC (1:1000, AG-25B-0006) antibodies were purchased from Adipogen (San Diego, CA, USA).

Mice

Male NOD/SCID/Il2rg null (NSG) mice 6 weeks old were obtained from GHbio (Daejeon,

South Korea, Nod/scid-IL2Rg^{em1}). Mice were kept under a 12:12 h light:dark cycle and specific pathogen-free conditions. Animal experiments were carried out in accordance with the relevant ethical guidelines.

Cells

Human PBMCs were prepared by using heparinized venous blood tubes (BD, 367874) and Lymphoprep™ (Alere technologies, Oslo, Norway). Blood samples were collected to heparinized venous blood tubes from healthy donors. PBMCs were isolated from blood within 3 h by density gradient centrifugation (800 × g, 20 min) without brake using Lymphoprep™ solution and cultured with RPMI-1640 medium containing 5% human serum (Sigma Aldrich, H3667) and 1% penicillin streptomycin amphotericin mixture (Lonza, 17-745E). Human primary monocytes were purified by using CD14 MicroBeads MACS kit (Miltenyi Biotech, Bergisch Gladbach, Germany, 130-050-201) and cultured with RPMI-1640 medium containing 5% human serum (Sigma Aldrich, H3667) and 1% penicillin streptomycin amphotericin mixture (Lonza, 17-745E). The cells were then incubated in medium containing 4 ng/mL human macrophage colony-stimulating factor (Sigma Aldrich, M6518) for 4 days to differentiate into human monocyte-derived macrophages for bacterial infection.

Murine BMDMs were collected by flushing bone marrow cells from the femurs and tibias of 6-8-week-old female C57BL/6 mice. The bone marrow cells cultured for 5-6 days in Dulbecco’s modified Eagle’s medium (DMEM; Corning, NY, USA, 10-013-CV) supplemented with 10% fetal bovine serum (FBS; Gibco, NY, USA, 16000-044) and 1% antibiotics (Lonza, 17-745E) containing 25 ng/mL macrophage colony-stimulating factor

(R&D Systems, MN, USA). The cells were washed with phosphate-buffered saline (PBS) and cultured in DMEM (10% FBS, 1% antibiotics) medium. Murine PMs were obtained from 6-8-week-old female C57BL/6 mice. For generation of PMs, mouse was administered an intraperitoneal injection of 1 ml of 3% thioglycolate (Sigma Aldrich, T9032). After 3 days, peritoneal fluid was harvested in ice-cold PBS containing 3% FBS. The PMs were collected from peritoneal fluid by centrifugation (500 × g, 10 min, 4 °C). After aspirating supernatant, the cell pellet was suspended with 1 – 2 ml red blood cell lysis buffer (Invitrogen, Waltham, MA, USA, 00-4333-57) for 5 min at 4 °C. The cells were washed twice with PBS and cultured in DMEM (10% FBS, 1% antibiotics) medium.

KG-1α, THP-1, and HL-60 cell lines were kindly provided by Prof. Jun Hyoung Heo (Chungnam National University, Daejeon, South Korea). KG-1α and THP-1 cells were cultured and split in RPMI-1640 (10% FBS, 1% antibiotics). HL-60 cell line was cultured and split in RPMI-1640 (20% FBS, 1% antibiotics). These cell lines were maintained at a density of 0.2–0.6 × 10^6 cells/mL in culture medium. K562-luc cell line was obtained from Prof. Song (Chungnam National University, Daejeon, South Korea). K562-luc cells were cultured and split in RPMI-1640 (10% FBS, 1% antibiotics). The cell line was maintained at a density of 0.3–0.4 × 10^6 cells/mL in culture medium.

Inflammasome analysis

Human PBMCs, monocytes were seeded into 48-well plates. After 16 h, we changed the culture media to fresh RPMI-1640 (5% human serum, 1% antibiotics). The cells were primed with 10 ng/mL LPS for 4 h, followed by the treatment with 10 μM nigericin (30 – 45 min), 5 mM ATP (30 – 45 min), MPXV A30L protein (18 h), or IAMP29 (18 h). THP-1

cells were seeded with 300 nM PMA into 48-well plates for 3 days. PMA-differentiated THP-cells were washed twice by PBS and replaced with fresh RPMI-1640 (5% FBS, 1% antibiotics). After resting for overnight, the cells were primed with 100 ng/mL LPS for 4 h, followed by the treatment with 10 μM nigericin (30-45 min), 5 mM ATP, MPXV A30L protein (18 h), or IAMP29 (18 h). BMDMs and PMs were seeded into 48-well plates. After 16 h, we replaced the culture medium with fresh Opti-MEM medium (gibco, Basel, Switzerland). The cells were primed with 100 ng/mL LPS for 4 h, followed by the treatment with 10 μM nigericin (30-45 min), 5 mM ATP, MPXV A30L protein (18 h), or IAMP29 (18 h).

Lentiviral shRNA preparation and knockdown of NLRP3 and PKM2

Lentiviruses were produced by transfecting HEK293T cells (ATCC, Manassas, VA, USA; CRL-11268) seeded the previous day in 6-well plates using a three-plasmid system. When the HEK293T cells reached 60–70% confluency, they were transfected with pLV[shRNA]-Puro-U6 plasmids targeting hNLRP3 (5'-GCGTTAGAAACACTTCAAGAA-3') and hPKM2 (5'-AGGCAGAGGCTGCCATCTACC-3') (VectorBuilder, Chicago, IL, USA), along with a third-generation lentiviral packaging system (pRSV-Rev, #12253; pMD2.G, #12259; and pMDLg/pRRE, #12251, obtained from Addgene). The viral supernatant was collected 52 h post-transfection. At 24 h post-transfection, the medium was discarded and replaced with DMEM containing 10% FBS. After 48 h, the supernatant was harvested and filtered using a 0.45-μm pore membrane filter (Merck Millipore, Burlington, MA, USA; SLHVR13SL). The filtered supernatant was then immediately used for lentiviral shRNA transduction in human primary monocytes over a period of 24–36 h.

RNA extraction and quantitative reverse transcription polymerase chain reaction (qRT-PCR)

Total RNA was extracted using TRIzol reagent (Invitrogen, 15596026). To synthesize cDNA, we used Reverse Transcriptase Premix (ELPIS Biotech, Daejeon, Korea, EBT-1515). SYBR Green Master Mix (Qiagen, Germantown, MD, USA; 204074) was used for qRT-PCR in the Rotor-Gene Q 2plex System (Qiagen). Analysis was conducted by the $2^{-\Delta\Delta Ct}$ method with normalization to *GAPDH* gene. Primer sequences are listed in Supplementary Table 1.

Western analysis

Cells were lysed in radioimmunoprecipitation assay (RIPA) buffer containing protease and phosphatase inhibitors. Proteins in the supernatant were precipitated and concentrated by incubating with StrataClean Resin (final concentration: 0.6% v/v; Agilent Technologies) for 1 minute at 4°C. The StrataClean Resin was then vortexed and centrifuged at 200 × g for 1 minute at 4°C. The proteins were eluted by resuspending the StrataClean Resin in 5 × SDS sample buffer. All protein samples were subsequently mixed with an equal volume of SDS sample buffer (Elipis Biotech, Korea) and boiled for 10 minutes.

SDS-polyacrylamide gel electrophoresis (SDS-PAGE) was employed to separate the proteins. The separated proteins were then transferred onto either nitrocellulose or polyvinylidene fluoride (PVDF) membranes (Millipore). To prevent non-specific binding, the membranes were immersed in 0.1% TBST for 30 minutes. The membranes were then incubated with specific primary antibodies followed by peroxidase-conjugated secondary antibodies. Signals were detected using ECL solution (Millipore) and visualized with the

appropriate detection equipment (iBright CL750 Imaging System, Invitrogen, A44535).

Enzyme-linked immunosorbent assay (ELISA)

Supernatants of PBMCs, monocytes, PMA-differentiated THP-1 cells, murine BMDMs, and PMs were stored at −80°C and analyzed for ELISA. The BD OptEIA Set ELISA Kits (BD Biosciences) for human TNF (555212), human IL1B (557953), and Human IL-18 ELISA (Invitrogen, KHC0181) were used for human PBMCs, human primary monocytes, and PMA-differentiated THP-1 cells. Mouse IL1B ELISA kit (Invitrogen, 88-7013-88) from was used for murine BMDMs, and PMs. The ELISA experiment and data analysis were performed according to the manufacturer’s protocols.

Confocal microscopy

The coverslips were incubated with Poly-L-lysine solution (PLL; Sigma Aldrich, P4832) for at least 2 h at room temperature. The coverslips were washed by PBS and culture media 2 times and dried for 1 h. The 24-well culture plate was seeded the 1×10^5 monocytes on a PLL-coated coverslip. Cells were stained with DAPI (Invitrogen, 00-4959-52) and incubated with anti-FLAG antibody (1:400 dilutions; P7425, Sigma Aldrich) for detecting the IAMP29 with FLAG tag. Following 3 - 5 washes in the PBS, the primary antibody binding was visualized by incubation with Alexa Fluor® 594 conjugate secondary antibody (1:400 dilutions; Thermo Fischer, A-11012) diluted in the washing solution for 2 h at room temperature.

To quantify mtROS, the human monocytes on the PLL-coated coverslip were loaded with 2 μM MitoSOX solution (Invitrogen, M36008) at 37°C for 30 min. After 30 min

incubation, the coverslips were washed by PBS and stained with DAPI (Invitrogen, 00-4959-52). The cell images were captured by using confocal microscope ZEISS LSM 900. MitoSOX intensity was analyzed with image J software (1.53v).

Flow cytometry

Pyroptosis was quantified by BD FACSCanto II (BD Bioscience) using the FITC Annexin V (BD Bioscience, USA) and propidium iodide (PI) (BD Bioscience, USA), following the manufacturer's instructions (BD Pharmingen™ FITC Annexin V Apoptosis Detection Kit I). In brief, after the IAMP29 treatment, cells were harvested and washed twice with cold PBS. Samples were incubated in 100 µL of binding buffer (BD Bioscience, USA) containing 2.5 µL Annexin V-FITC and 1 µL PI at 4°C for 15 min in the dark. After incubation, 400 µL of binding buffer was added, and cell pyroptosis was detected through flow cytometry. Stained cells were analyzed using FlowJo software version 10.0.7 (BD Biosciences).

Immunoprecipitation (IP) and IP-based LC-tandem mass spectrometry (IP-MS)

Cells were lysed in cold M2 lysis buffer (20 mM Tris [pH 7.6], 0.5% NP-40, 250 mM NaCl, 3 mM ethylenediaminetetraacetic acid [EDTA], 3 mM egtazic acid [EGTA], 0.5 mM phenylmethyl sulfonyl fluoride, 20 mM β-glycerol phosphate, 1 mM sodium vanadate, and 1 µg/mL leupeptin). Fractionated lysates (1-2 mg) were incubated overnight with 20 µL of Anti-Flag® M2 Affinity Gel (Millipore, A2220) at 4°C with gentle shaking, followed by four washes with cold M2 buffer to isolate immunocomplexes. Immunocomplexes were fractionated by 4-12% SDS-PAGE (Invitrogen) and subjected to Western blotting using

PKM1 (Cell Signaling Technology, Danvers, MA, USA, #7067) or PKM2 (Cell Signaling Technology, #4053) antibodies, or visualized by staining with InstantBlue® Coomassie Protein Stain (Abcam, Cambridge, UK, ab119211) for liquid chromatography–tandem mass spectrometry (LC-MS/MS). Stained gel lanes were divided based on molecular weight; sliced gels were destained with acetonitrile (Sigma Aldrich, 271004), reduced with dithiothreitol (Sigma Aldrich, D9779), alkylated with iodoacetamide (VWR, Radnor, PA, USA, 97064-926), and digested with trypsin (Promega, Madison, WI, USA, V5280). Digested peptides (5 μl) were concentrated and separated in a Nanoviper Column (Thermo Fisher Scientific). MS and MS/MS spectra were acquired using a Q Exactive Plus Ion-Trap Mass Spectrometer in data-dependent mode. For protein identification, nano LC-MS/MS spectra were searched using MASCOT version 2.7 (Matrix Science) based on data from the UniProt human data resource.

Extracellular acidification rate (ECAR) analyses

After stimulation with LPS (10 ng/mL) for 2 h, PMA-differentiated THP-1 cells were seeded at 3 × 10^4 per well in an XF96 cell culture microplate, incubated overnight at 37°C, and treated with vehicle or IAMP29 (5 μg/mL) for 18 h. Prior to analysis, 180 μL of assay medium (XF base medium containing 1 mM L-glutamine [pH 7.4]) were added to each well, and the plate was incubated for 1 h at 37°C in an incubator without CO_2. Agilent Technologies XF Calibrant Solution (1 mL/well) was used to activate the XF96 Biosensor Cartridge for 24 h at 37°C in an incubator without CO_2. The basal ECAR was measured in glycolysis stress-test medium, and sequential injections of the following reagents were performed: 100 mM glucose (to fuel glycolysis, final concentration 10 mM), 100 μM

oligomycin (to inhibit adenosine triphosphate [ATP] synthase in mitochondria, 10 μM), and 500 mM 2-deoxyglucose (for competitive inhibition of glucose, 50 mM).

Mycobacterial strains and cultivation

M. bovis Bacillus Calmette Guérin (BCG; ATCC35734) and smooth morphotype of *M. abscessus* subsp. abscessus (Mabc; ATCC19977) used in this study were obtained from American Type Culture Collection (ATCC). Clinical strain smooth morphotype of *M. abscessus* subsp. *bolletii* (Mboll; KMRC-00800-00023) used in this study was kindly provided by Dr. J Whang (Korean Mycobacterium Research Center). All BCG, Mabc, and Mboll strains were cultured in Middlebrook 7H9 (Difco, Sparks, MD, 271310) medium supplemented with 10% oleic albumin dextrose catalase (OADC; BD Biosciences, San Diego, CA, 212240), 0.5% glycerol (Sigma-Aldrich), and 0.05% Tween-80 (Sigma-Aldrich) on a rotary shaking incubator (140 rpm) at 37°C to an OD_{600} of 0.4-0.6. The harvested pellets of cultures were washed out at least three times with PBS supplemented with 0.05% Tween-80 by centrifugation at 3,000 rpm for 10 min at 4°C. Final pellets were resuspended in PBS, aliquoted, and stored at -80°C. The randomly selected three vials were thawed and used to CFUs on Middlebrook 7H10 agar (Difco, 262710).

Bacterial infection and colony-forming units (CFU) assay

Human monocyte-derived macrophages (1×10^4 cells/well) were seeded in 96 well plate. Murine peritoneal macrophages (2×10^5 cells/well) in a 48-well plate. Both cell types were infected with thawed Mboll (MOI 1) or Mabc (MOI 1) bacterial cells from -80°C freezer for 2 h at 37°C in a humidified atmosphere of 5% CO_2. After infection, extracellular bacteria

were washed out with warmed PBS, and the infected cells were further cultured within freshly changed medium containing 10 μg/mL gentamicin for 24 or 48 h. To evaluate the viability of intracellular bacteria, infected cells were lysed in distilled water. The cell lysates were collected, serially diluted, and then plated on Middlebrook 7H10 agar, and incubated for 3-5 days. Colonies were enumerated to compare the CFU values between control and experimental groups.

Animal experiments with tumor xenograft and analysis of tumor burden using an in vivo imaging system platform

K562-luc cells (4×10^6 in 100 μL of Hank's balanced salt solution) were intravenously injected into the tail vein of NOD/SCID/Il2rg null mice (Koatech, South Korea). IAMP29 (36 mg/kg) was administered intraperitoneally every other day, starting 7 days after the confirmed engraftment of K562-luc cells. Tumor burden was assessed using the IVIS system once a week for up to 4 weeks following the transplantation of K562-luc cells.

The IVIS platform used to measure the tumor burden was as follows: Mice were injected intraperitoneally with 150 mg/kg body weight of IVISbrite D-luciferin potassium salt, a bioluminescent substrate (PerkinElmer #122799) for luciferase. After being anesthetized with 1.5% isoflurane and 100% O2, the mice were positioned in the abdominal position on the imaging stage of the IVIS apparatus. Images were captured using the IVIS Imaging system, specifically the Lumina XRMS instrument by PerkinElmer, within 10 minutes after the injection with luciferin. The Aura imaging software (Spectral Instruments Imaging) was used to measure photons emitted from the tumor and its surrounding areas.

Poly(D,L-lactic-co-glycolic acid) nanoparticle (PLGA NP) production and characterization

PLGA NPs were acquired from Nanoglia (Daejeon, South Korea). All reagents and solvents were of analytical grade. To create IAMP29-encapsulated PLGA NPs, 182 mg/mL of IAMP29 in 200 μL distilled water was gradually added to 0.8 mL of dichloromethane containing 2.5 mg of PLGA, then emulsified by sonication to form a primary W1/O emulsion. Subsequently, 2 mL of 1% (w/v) PVA1500 (Alfa Aesar, Ward Hill, MA, USA, 041243) were introduced, and the mixture was emulsified by sonication for 1 min to form a W1/O/W2 double emulsion. Next, 6 mL of 1% (w/v) PVA1500 were added, and the dichloromethane was evaporated by magnetic stirring for 3 h at room temperature in a fume hood. PLGA NPs were harvested by centrifugation at 13,000 rpm for 10 min at 4°C, washed twice with deionized water, and freeze-dried. The NPs were diluted in double-distilled water for size and zeta potential analyses by dynamic light-scattering assays using the Zetasizer Nano ZS90 (Malvern Panalytical, Worcestershire, UK).

Supplementary Fig. 1

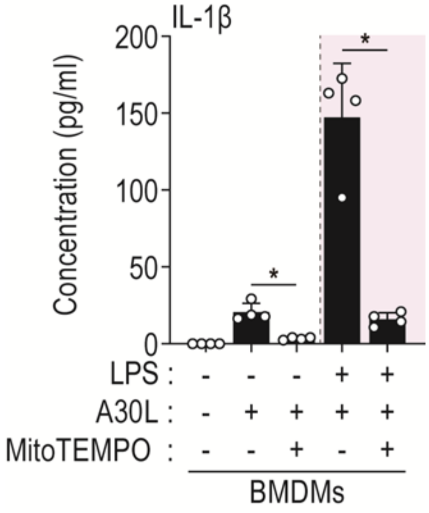

Supplementary Fig. 1. A30L induces the production of IL-1β via generation of mtROS in murine macrophages. IL-1β concentration in the culture supernatants of A30L-treated BMDM with the pretreatment of MitoTEMPO. The BMDM were treated 40 μg/mL A30L for 18 h after 2 h pretreatment of 100 μM MitoTEMPO. Statistical analysis was conducted with an unpaired *t*-test with Mann-Whitney test or a one-way ANOVA with Tukey's multiple comparison test and presented as means ± SD. *p < 0.05; **p < 0.01; ***p < 0.001; ****p < 0.0001. ns, not significant.

Supplementary Fig. 2

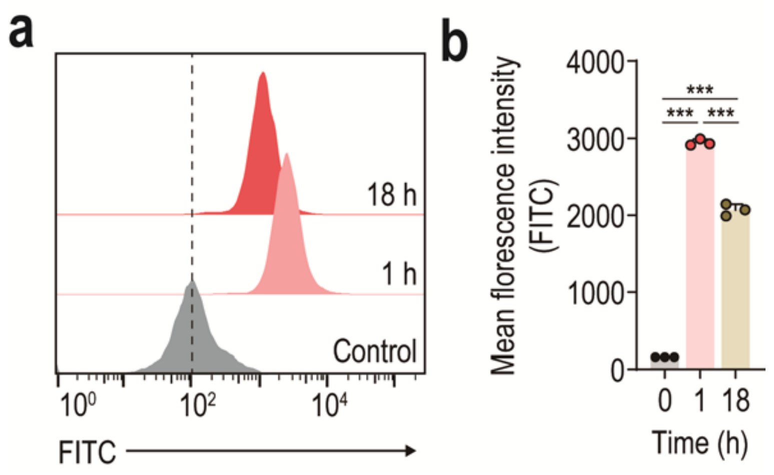

Supplementary Fig. 2. FITC-labelled IAMP29 is internalized into human primary monocytes. a, b Human monocytes were incubated 1 h or 18 h with 25 µg/mL IAMP29. The mean fluorescence intensity was detected by flow cytometry. Statistical analysis was conducted with a one-way ANOVA with Tukey's multiple comparison test and presented as means ± SD. *p < 0.05; **p < 0.01; ***p < 0.001; ****p < 0.0001.

Supplementary Fig. 3

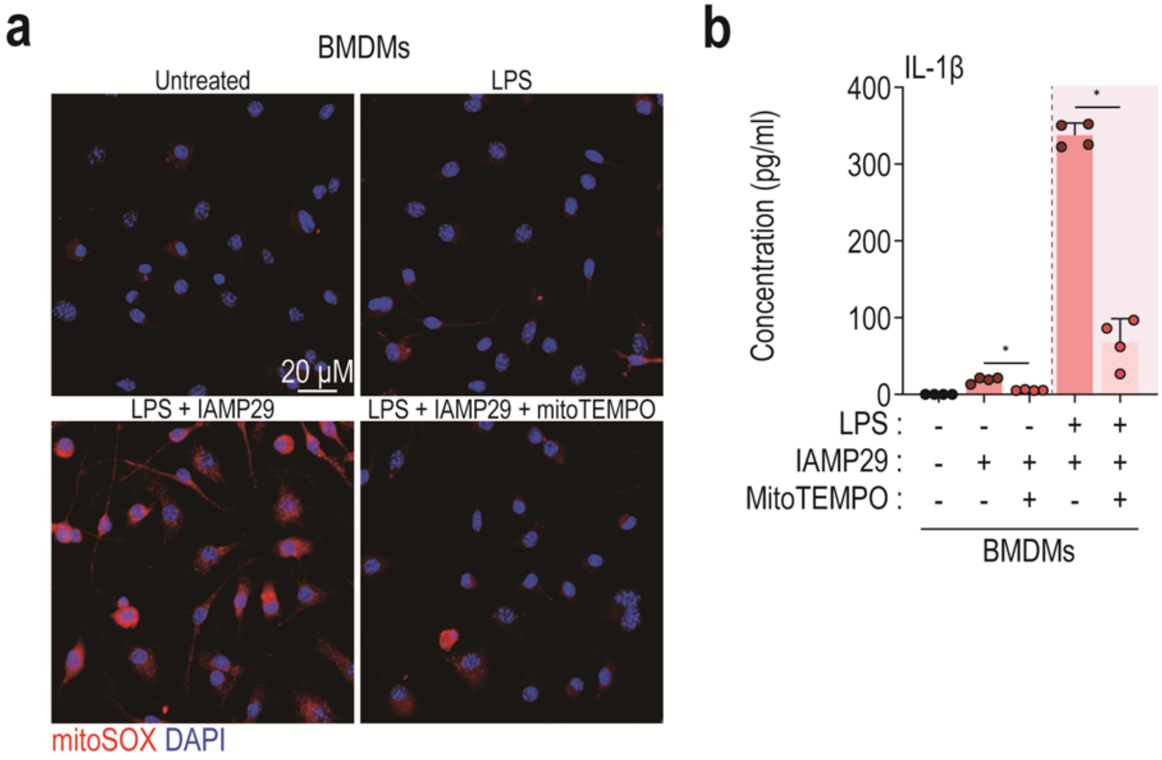

Supplementary Fig. 3. IAMP29 induces the production of IL-1β via generation of mtROS in murine BMDMs. a Representative immunofluorescence images (scale bar = 20 μm) of relative fluorescence intensities of DAPI versus mtROS. Murine BMDMs were treated with IAMP29 (80 μg/mL for 4 h) after pretreatment of MitoTEMPO (100 μM for 2 h). The mtROS and nuclei were stained with MitoSOX Red (red) and DAPI (blue), respectively. **b** IL-1β concentration in the culture supernatants of IAMP29-treated BMDM (80 μg/mL for 18 h) with the pretreatment of MitoTEMPO (100 μM for 2 h). Statistical analysis was determined by Mann–Whitney U test. *$p < 0.05$; **$p < 0.01$; ***$p < 0.001$.

Supplementary Fig. 4

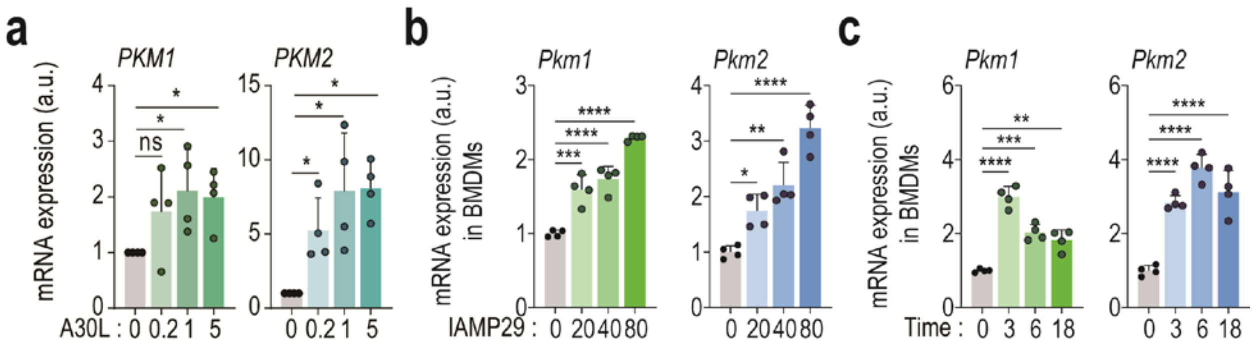

Supplementary Fig. 4. Transcriptional activation of PKM in immune cells by A30L and IAMP29. a mRNA expression levels of *PKM1* and *PKM2* in A30L-treated human primary monocytes. The monocytes were treated with A30L in dose-dependent manner. **b, c** mRNA expression levels of *Pkm1* and *Pkm2* in IAMP29-treated murine BMDMs. The murine BMDMs were treated with IAMP29 in dose (**b**) or time (**c**)-dependent manner. Statistical analysis was determined by a one-way ANOVA with Tukey's multiple comparison test. $^{*}p < 0.05$; $^{**}p < 0.01$; $^{***}p < 0.001$. a.u., arbitrary unit;

Supplementary Fig. 5

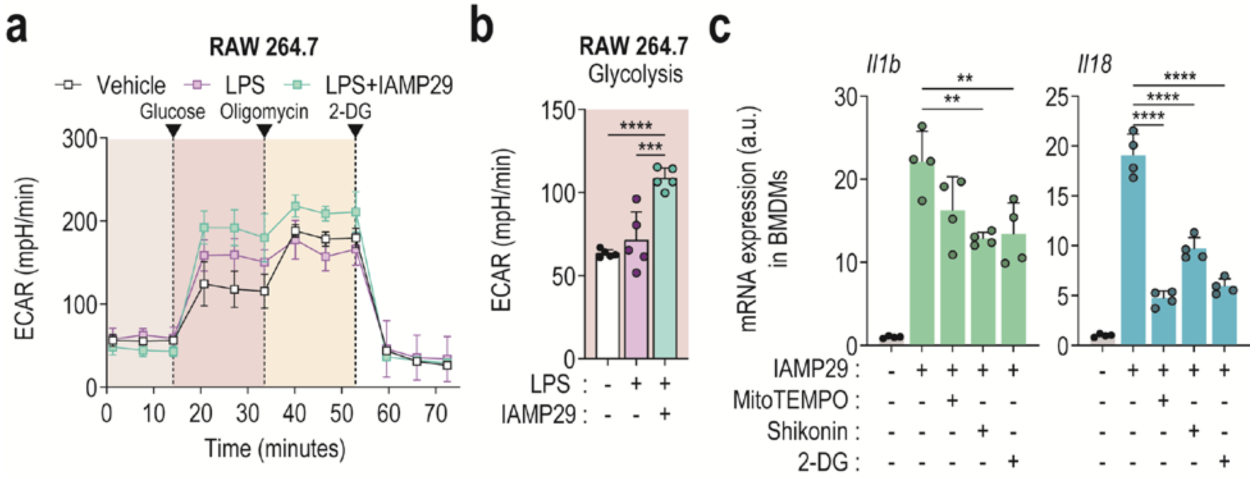

Supplementary Fig. 5. Inhibition of IAMP29-induced glycolysis suppresses Signal 1 activation in murine macrophages. a, b The level of ECAR in RAW 264.7 cells with IAMP29 (40 µg/ml, 18 h) after LPS stimulation. **c** The mRNA expression levels of *Il1b* and *Il18* in IAMP29-treated murine BMDMs after pretreatment of indicated inhibitors. Statistical analysis was determined by a one-way ANOVA with Tukey's multiple comparison test and presented as means ± SD. *p < 0.05; **p < 0.01; ***p < 0.001; ****p < 0.0001.

Supplementary Fig. 6

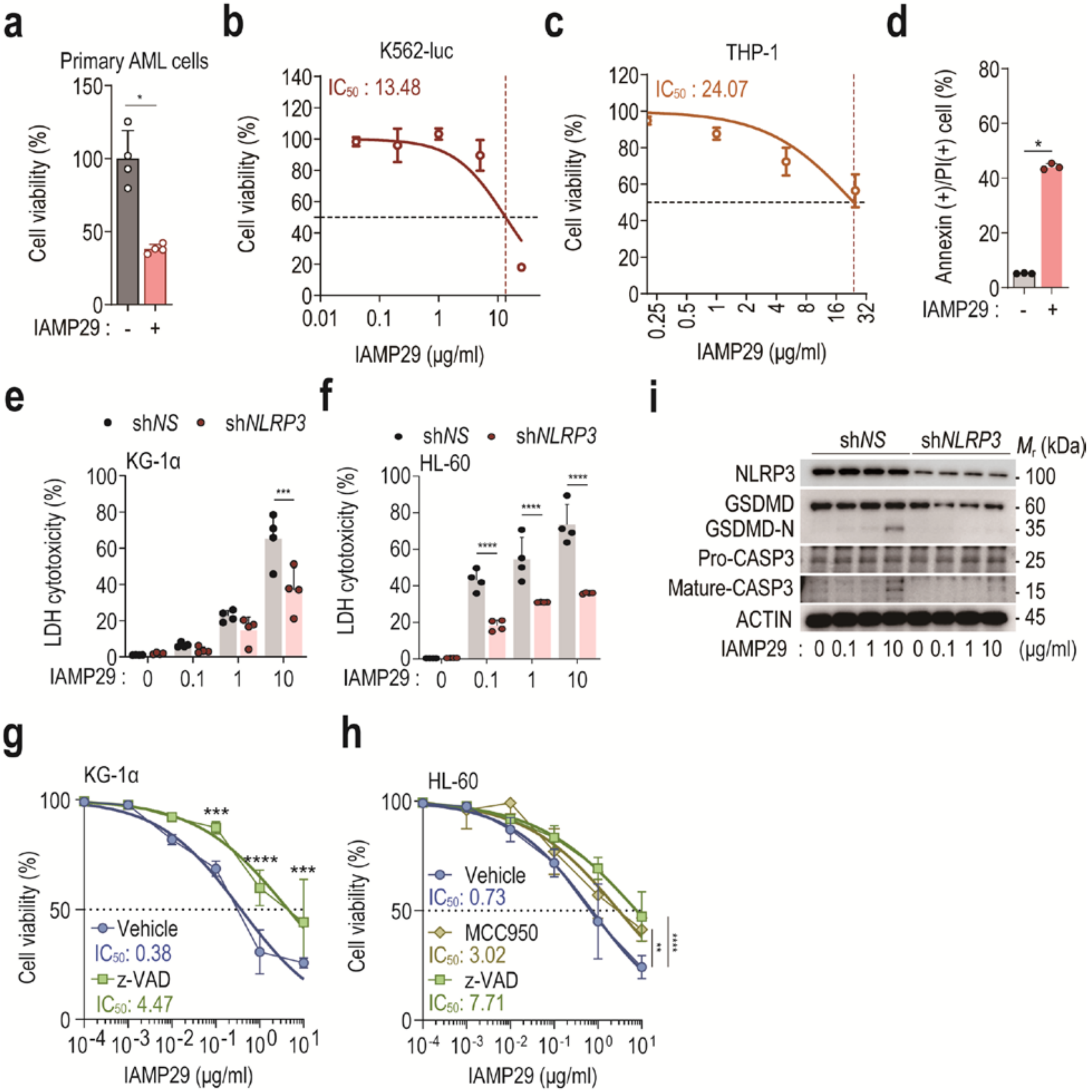

Supplementary Fig. 6. IAMP29 treatment activates both apoptotic and pyroptotic responses in human leukemic cells. a-c The cytotoxic effect of IAMP29 in patient-

derived AML cells (24 h), human K562-luc (48 h), and THP-1 (24 h), assessed by CCK-8 assay. **d** The graph showing the percentage of Annexin V/PI-stained HL-60 cells with or without IAMP29 treatment (2 μg/mL for 24 h). **e, f** The cytotoxic effect of IAMP29 in KG-1α (**e**) or HL-60 cells (**f**) with/without NLRP3 knockdown, assessed by LDH assay. **g, h** The cytotoxic effect of IAMP29 with the presence or absence of indicated inhibitors in KG-1α (**g**) or HL-60 cells (**h**). **i** Western blotting for GSDMD, cleaved GSDMD (GSDMD-N), Caspase-3, and mature caspase-3 in cell lysates of IAMP29-treated HL-60 cells with NLRP3 knockdown. Statistical analysis was determined by a one-way ANOVA with Tukey's multiple comparison test and presented as means ± SD. *$p < 0.05$; **$p < 0.01$; ***$p < 0.001$; ****$p < 0.0001$.

Supplementary Fig. 7

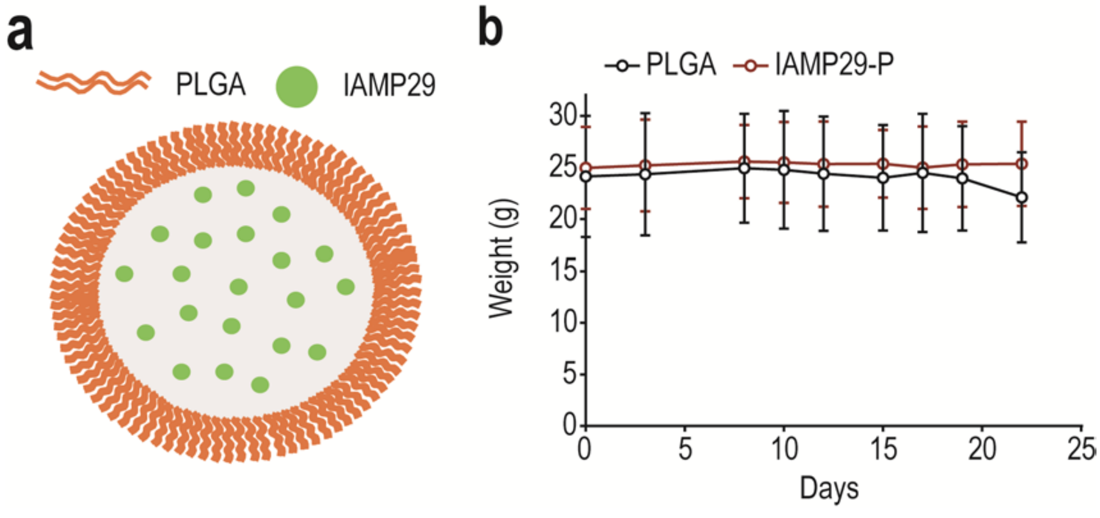

Supplementary Fig. 7. PLGA-encapsulated IAMP29 treatment does not affect the body weight of mice. a Schematic overview of the IAMP29-encapsulated PLGA NPs studied. **b** Body weight of mice in PLGA- or IAMP29-P-treated mice. Statistical analysis was determined by two-way ANOVA with Bonferroni's multiple comparisons test and presented as means ± SD from at least four biological replicated experiments performed.

Supplementary Table 1. Primers used for qRT-PCR in this study.

Genes	Species	Primer	Sequences (5’ – 3’)
IL1B	*Homo sapiens*	Forward	CGCCAGTGAAATGATGGCTTA
		Reverse	TGCTGTAGTGGTGGTCGGA
IL18	*Homo sapiens*	Forward	CCACCACAAGGCGCTGTTCTACA
		Reverse	AGGCGGAGGACTCGAACTCGTA
IL15	*Homo sapiens*	Forward	GCTGGAAACCCCTTGCCATA
		Reverse	CTGCACTGAAACAGCCCAAAA
CX3CL1	*Homo sapiens*	Forward	GCAAACGCGCAATCATCTTG
		Reverse	GTGCCGCCATTTCGAGTTAG
CXCL10	*Homo sapiens*	Forward	TCCTGCAAGCCAATTTTGTCC
		Reverse	TGTGGTCCATCCTTGGAAGC
PKM1	*Homo sapiens*	Forward	CTATCCTCTGGAGGCTGTGC
		Reverse	CGCACAAGTTCTTCAAACAGC
PKM2	*Homo sapiens*	Forward	CTATCCTCTGGAGGCTGTGC
		Reverse	GTGGGGTCGCTGGTAATG
GAPDH	*Homo sapiens*	Forward	CAAGATCATCAGCAATGCCTCC
		Reverse	GGTCATGAGTCCTTCCACGA
Pkm1	*Mus musculus*	Forward	GCTGTTTGAAGAGCTTGTGC
		Reverse	TTATAAGAGGCCTCCACGCT
Pkm2	*Mus musculus*	Forward	TCGCATGCAGVACCTGATT
		Reverse	CCTCGAATAGCTGCAAGTGGTA
Hif1a	*Mus musculus*	Forward	CAAGATCTCGGCGAAGCAA
		Reverse	GGTGAGCCTCATAACAGAAGCTT
Il1b	*Mus musculus*	Forward	TGACGGACCCCAAAAGATGA
		Reverse	AAAGACACAGGTAGCTGCCA
Il18	*Mus musculus*	Forward	CAACTTTGGCCGACTTCACTG
		Reverse	TCAGTCTGGTCTGGGGTTCA
Actb	*Mus musculus*	Forward	CCACCATGTACCCAGGCATT
		Reverse	AGGGTGTAAAACGCAGCTCA
